# Inadequate housing and pulmonary tuberculosis: a systematic review

**DOI:** 10.1186/s12889-022-12879-6

**Published:** 2022-03-30

**Authors:** Ju-Yeun Lee, Namhee Kwon, Ga-yeon Goo, Sung-il Cho

**Affiliations:** 1 The Department of Public Health, Graduate School of Public Health, Building 220, Seoul National University, 1 Gwanak-ro, Gwanak-gu, Seoul, 08826 Republic of Korea; 2 The Department of Health Care Management and Policy, Graduate School of Public Health, Seoul National University, Seoul, Republic of Korea; 3 The Department of Public Administration, Graduate School of Public Administration, Seoul National University, Seoul, Republic of Korea; 4 Graduate School of Public Health and Institute of Health and Environment, Seoul National University, Seoul, Republic of Korea

**Keywords:** Tuberculosis (TB), Housing, Housing affordability, Housing quality, Homelessness, Systematic review

## Abstract

**Background:**

Tuberculosis (TB) is a global health issue that has long threatened and continues to threaten human health. While previous studies are important in the search for a cure for TB, to eradicate the disease it is also crucial to analyze environmental influences. Therefore, this study determined the potential effect of inadequate housing on TB and the magnitude of the effect.

**Methods:**

This is a systematic review of the effects of inadequate housing on TB. Between Jan 1, 2011 and Oct 25, 2020, we searched four electronic databases using the search terms “housing AND tuberculosis” or “housing AND TB”. The target population comprised residents of inadequate housing and the homeless.

**Results:**

We found 26 eligible studies. The distribution of the studies across continents was uneven, and the housing issues of interest seemed to vary depending on the economic level of the country. The eight steps identified in TB development and the consequences thereof were more strongly associated with housing affordability than with housing quality.

**Conclusions:**

This is the first systematic review to identify the effects of inadequate housing on TB and to categorize inadequate-housing-related exposure to TB in terms of affordability and quality. The steps identified in TB development and the consequences thereof had a greater association with housing affordability than with housing quality. Therefore, public health interventions regarding housing affordability could be more diverse, and interventions that support affordable housing for residents of inadequate housing and the homeless should proceed simultaneously to improve housing quality.

**Supplementary Information:**

The online version contains supplementary material available at 10.1186/s12889-022-12879-6.

## Introduction

Tuberculosis (TB) has long been a threat to global health and was the leading cause of death from a single infectious agent before the COVID-19 pandemic. The World Health Organization (WHO) announced that in 2020 there were 5.8 million cases of TB and 1.5 million deaths worldwide [1]. Because of the pandemic, the number of people newly diagnosed with TB decreased in 2020, while the number of TB deaths increased compared to 2019 [1]. The cumulative rate of decrease in TB infections between 2015 and 2020 was 11% [1]. Although the global incidence of TB is declining, this rate of decrease is insufficient. The numbers of new or relapsed cases range from < 5 to > 500 per 100,000 of the population per year depending on the country [1], and therefore TB remains a major challenge to global health and societal costs.

TB has been used as a typical example of a “social disease” [2], but the causal pathway of the socioeconomic development and epidemiology of TB has not been fully described. Major socioeconomic factors that influence vulnerability to ill health include housing, poverty, gender, religion, education, the workplace, lifestyle, and the health care system [3]. Socioeconomic elements that negatively affect TB include inadequate housing with overcrowding and poor ventilation, malnutrition, alcohol abuse, smoking, limited education, poor quality of life, low income, and unemployment [1, 4–8]. To fully control TB, it is necessary to examine societal risk factors of the disease, in particular the main socioeconomic risk factor for TB: inadequate housing.

Housing is a precondition for enjoying several human rights [9]. The United Nations declared adequate housing “key for development and social equality” as Goal 11 of the Sustainable Development Goals (SDG). Inadequate housing is associated with TB, with TB syndemics (typically HIV), and with TB comorbidities (e.g., diabetes and alcohol abuse). The WHO End TB Strategy, included in SDG Goal 3, stipulates the need for action on social protection, and adequate housing equals social protection. Based on the housing conditions defined as adequate by the UN International Covenant on Economic, Social and Cultural Rights, inadequate housing means unmet needs for freedoms and entitlements, not just four walls and a roof [9]. Therefore, adequate housing must encompass more than a building, including security of tenure, location, cultural adequacy, availability of services, materials, facilities and infrastructure, habitability, accessibility, and affordability [10]. In this context, inadequate housing includes not only poor-quality housing but also limited affordability of adequate housing.

Housing quality has been defined for residential environments (e.g., crowding, ventilation, dampness). Here we define housing affordability as the ability to choose and maintain a living space with an appropriate cost burden that does not threaten or undermine the occupant’s enjoyment of other human rights according to the United Nations right to adequate housing [9]. Although there is no universal definition of this term, housing affordability refers to “the cost of housing services and shelter relative to a given individual’s or household’s disposable income” [11]. In general, it is considered appropriate when a family (including a one-person family) spends less than 30% of its income renting or buying a house [12]. Although housing instability (e.g., moving frequently or couch surfing) is sometimes distinguished from affordability [13], we define housing affordability, including housing instability, as rent strain that can overlap in terms of instability and affordability.

As emphasized in the WHO End TB Strategy, three pillars must work together to eradicate TB. These pillars are integrated, patient-centered TB care and prevention; intensified research and innovation; and bold policies and supportive systems [14]. Biomedical approaches eventually revert to the need for social, economic, and environmental interventions [7]. For example, drug-resistant TB is a primary challenge in the eradication of TB and is the result of failure to complete treatment; one of the main reasons for treatment failure is economic [15]. In poor countries, unemployment, low annual incomes, and loss of daily wages may lead to nonadherence to TB treatment [16, 17]. Approximately 49% of TB patients and their families bear the catastrophic costs of TB [1], and the social implications of these costs are linked to the proximal risk factors for TB [18], including inadequate housing.

Studies have considered housing and health frameworks [13, 19] and proximal risk factors for TB [7, 8] as the effects of housing on TB development, but the consequences have not been explained fully. Therefore, we examined associations between TB and housing in a housing and health framework and linked them to proximal risk factors for TB. Although the importance of housing as a social determinant of health is clear, many studies have focused only on host risk factors. Researchers have found associations between various aspects of housing and health, but studies of the negative effects of inadequate housing on respiratory health typically focus on asthma [13, 19], not TB. The framework for proximal risk factors for TB focuses on inadequate housing (e.g., crowding, poor ventilation) as a risk factor for TB exposure [7, 8].

Among the effects of inadequate housing on TB, the effects of overcrowding are the best known. Overcrowded housing leads to TB exposure [2, 7] and is a risk factor for the transmission [1, 18] and incidence [1] of TB. In addition to overcrowding, the indoor quality of housing, such as ventilation and dampness [7], is a risk factor for TB. Many studies have examined TB in the homeless, but few have considered the effects of housing affordability on TB. Inadequate housing, an environmental factor strongly associated with TB, has long been a global health issue, but few studies have identified the effects of inadequate housing on TB, and no systematic studies have been conducted. Identification of specific TB risk factors related to housing could help to identify strategies for combatting TB. Therefore, we conducted a systematic review of aspects of housing that are important social components of the eradication of TB.

## Methods

### Search strategy and study selection

This systematic review examined the effects of inadequate housing on TB, following the PRISMA 2020 guidelines [20] (Additional file 5: Appendix 1). Between Jan 1, 2011 and Oct 25, 2020, three reviewers (JYL, NK, and GG) searched four electronic databases (PubMed, EMBASE, ScienceDirect, and Web of Science) using the search terms “housing AND tuberculosis,” or “housing AND TB.” In the process of selecting search terms, the reviewers considered “housing”, “dwelling”, “settlement”, “residence”, and “homelessness”. The reviewers excluded studies of homelessness that leaved out other aspects of status (e.g., street homelessness, shelter homelessness) or that lacked non-homeless comparators because they were not within the scope of this study. The reviewers also searched the references of eligible studies to find other relevant studies. Four reviewers (JYL, NK, GG, and SIC) developed the review protocol, specifying the search strategies and exclusion criteria in advance (Additional file 5: Appendix 2).

Duplication of studies was identified with EndNote, and any duplicates not identified by the bibliography program were excluded after a review of the full text. After excluding duplicates, the three reviewers conducted preliminary screening to determine whether the title and abstract met the inclusion criteria. Then the screened articles were identified for eligibility through a full-text review; the reviewers agreed on the studies to include based on consensus. Data were extracted independently by the three reviewers and cross-checked by all three. The selected articles and extracted data were verified by a fourth reviewer (SIC).

### Selection criteria

The inclusion criteria for this study were original articles, with the full text published in English, pertaining to research on housing and TB development and its consequences. Irrespective of study design, any studies that did not include details of housing were excluded. To focus on the results of recent studies and to facilitate direct comparisons by limiting the range of the research period, we also excluded articles published before 2000. The target population comprised residents of inadequate housing and the homeless; there were no specific criteria regarding other demographic characteristics.

### Data extraction

The following six categories of data were extracted from the final selected studies (see Additional files 1, 2):

1. study characteristics: continent, country and region, study design, study period, data source, and the target and comparison populations
2. publication details: first author and reference number
3. exposure variables: housing affordability including homelessness, overcrowding, and housing quality
4. definitions of housing affordability and quality
5. outcome range among the target populations: all reported measures of point estimates were included with each measure of precision [95% confidence interval] in the comparison groups; and
6. study outcome: steps in TB development and the consequences thereof.
7. Based on existing studies [8, 14, 21, 22], we classified TB development and its consequences into the following eight steps from the perspective of TB epidemiology: exposure, detection, incidence, transmission, treatment adherence, drug resistance, treatment success, and recurrence. The target population of each study was divided according to the type of housing (shared housing, homeless, or public rental housing), population recruitment area (community or hospital), patient characteristics (drug resistant, latent TB, or history of TB), treatment completion, and social service intervention.

### Risk of bias in each study

The full-text articles were independently assessed for quality by two reviewers (JYL and NK) using the Joanna Briggs Institute (JBI) checklists [23], which have proven useful in systematic reviews encompassing different study designs. Each checklist was classified differently according to the study design (i.e., cohort, cross-sectional, or case–control). The quality of three ecological studies included in the selected studies could not be assessed because they lacked a specific checklist. Discrepancies were resolved through discussion to arrive at a consensus (JYL and NK) or by consultation with the fourth reviewer (SIC). The risk of bias in the 23 studies was classified as low, intermediate, or high. The risk criteria for each grade were 80–100% (low), 50–79% (intermediate), and 0–49% (high) for the percent “yes”. Of the selected studies, 21 had low risk and 2 had intermediate risk (see Additional file 4: Table S4_1). Cohen’s kappa for the included full-text articles was 1.0, indicating excellent agreement (range: 0.81–1.0) [24].

## Results

**Fig. 1:**
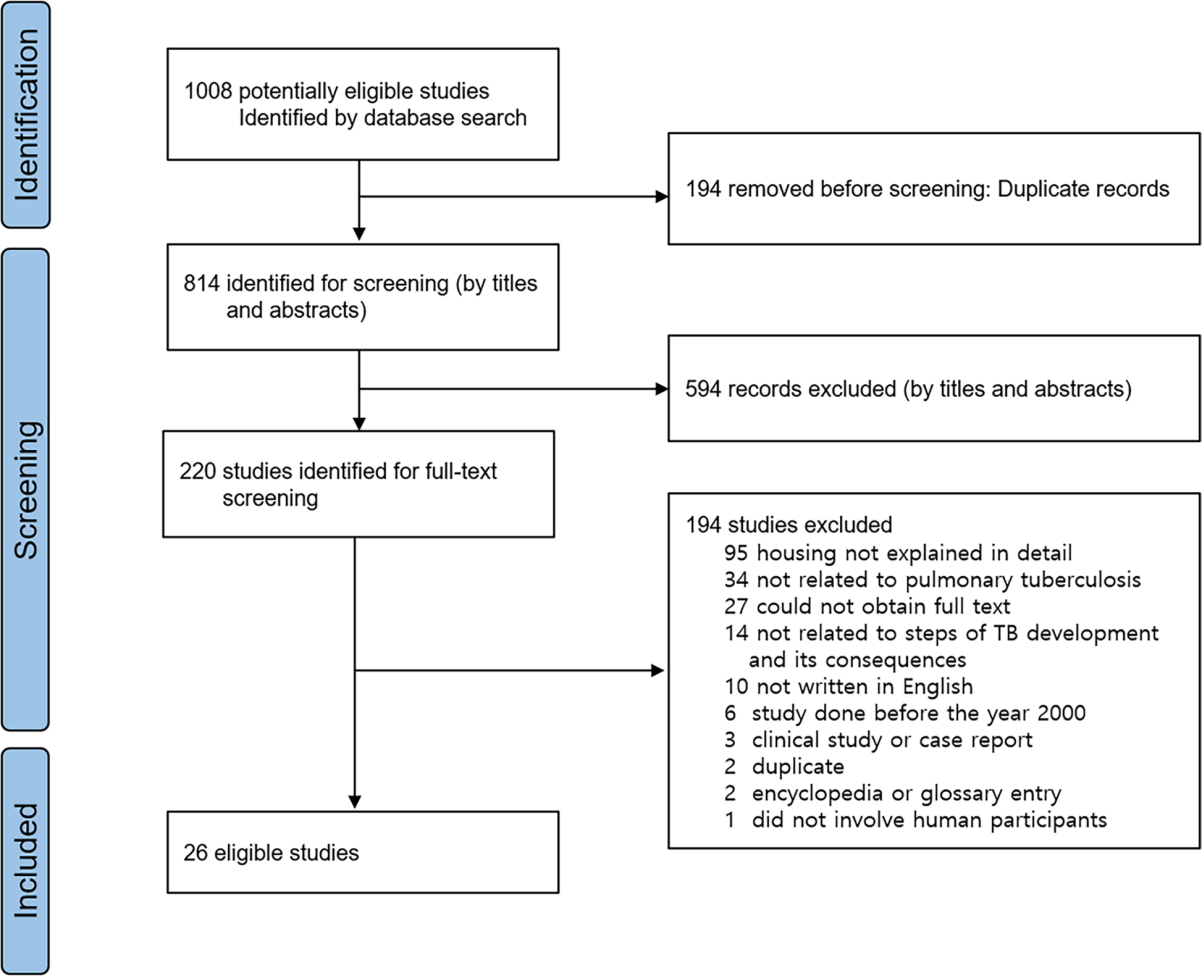
Selection of eligible studies

Among 1008 studies of housing and TB, we screened the titles and abstracts of 814 articles, after excluding duplicates. Of these screened studies, the full text of 220 was read by all researchers, from which 26 eligible studies were selected. Selection of the eligible studies accorded with the PRISMA 2020 flow diagram format (Fig. 1), resulting in exclusion of 194 articles (see Additional file 3).

The most common reason for ineligibility after the full-text review was the lack of a detailed explanation of the housing situation, such as no definition of poor housing conditions. Studies on verification or development of diagnostic methods were excluded as not being related to the steps in TB development and the consequences thereof.

Among the studies selected, there were 11 from Asia, the highest number among the continents, followed by 9 from the Americas, 5 from Africa, and 1 from Europe. The study distribution among the continents was uneven, and the housing issues of interest varied depending on the economic level of the country. Treatment adherence was the main focus of studies conducted in high-income countries such as the United States [25–28] and United Kingdom [29]. Overcrowded housing appeared to be a problem in low- and middle-income countries in Africa or Asia and in low-economic regions of high-income countries, whereas poor housing affordability is a global problem and coincides with homelessness. Studies of homelessness and TB were conducted only in South Korea [30,31, 32] and the United States [26, 27, 33–35] and mainly showed the effects of homelessness on TB prevalence [27, 31, 35] and treatment adherence [27, 30, 32], but homelessness also affected the detection rate [33] and incidence of TB [34]. The association between inadequate housing and TB discussed in each study is summarized according by continent (Table 1; see Additional file 1 for details).

**Table 1:** The association between inadequate housing and tuberculosis by continent

	Country	Study design	Summary
**Asia**			
Heo et al. (2012) [30]	South Korea	Cross-sectional	Among homeless TB patients, the type of residence and comorbidity with risk factor showed independent association with treatment success.
Haider et al. (2013) [36]	Pakistan	Case-control	The significant factors related to pulmonary TB status were daily contact with pulmonary TB patients and poor housing affordability.
Lai et al. (2013) [37]	Hong Kong	Ecological	Residence on lower floors in the dense clusters of high-rises are more prone to TB infection due to poorer air quality and lesser exposure to direct sunlight.
Lee et al. (2013) [31]	South Korea	Cross-sectional	The prevalence of LTBI among homeless was approximately five times higher than that of the non-homeless population and the TB burden among homeless should be managed by appropriate strategies.
Low et al. (2013) [38]	Hong Kong	Ecological	There was a strong negative relationship between TB outcome and floor levels of residence, and TB was more prevalent in public housing development.
Choi et al. (2016) [39]	South Korea	Cohort	Low educational levels, poor housing and occupations in the construction and manufacturing industries and service sectors were associated with poor treatment adherence.
Irfan et al. (2017) [40]	Bangladesh	Case-control	Significantly associated determinants of adult TB were over-crowding in a house, contact with a TB patient during the last 6 months and employed participants.
Rao et al. (2018) [41]	India	Cross-sectional	The socioeconomic risk factors such as malnutrition, poor living conditions in a kaccha house and tobacco smoking were significantly associated with pulmonary TB.
Kim et al. (2019) [32]	South Korea	Case-control	The housing provision package proved to be positively influential on the treatment outcomes of homeless TB patients that were grouped into COM, TAU and FAC
Saqib et al. (2019) [42]	Pakistan	Cross-sectional	The risk factors of household size, house structure, rooms in the home and room ventilation were significantly associated with TB history in patients.
Wardani et al. (2019) [43]	Indonesia	Case-control	The factors such as less ventilation, no in-house sunlight, existence of in-house smoking pollution and in-house TB contact significantly influenced on the TB infection risks.
**America**			
Kerker et al. (2011) [35]	USA	Cohort	The study aimed to systematically characterize the health of the population who used the New York city family shelter system, and on some level, higher risks posed by homeless children were the results of poverty and unstable housing with poor quality.
Bamrah et al. (2013) [26]	USA	Cohort	For homeless persons, TB incidence was 10 times higher and treatment incompletion was 2 times higher compared to those of the general population.
Feske et al. (2013) [27]	USA	Case-control	TB rates among the homeless were due to social determinants and homeless patients were hospitalized more days than the housed.
Hirsch-Moverman et al. (2015) [28]	USA, Canada	Cohort	The factors associated with LTBI treatment non-completion were severe symptoms, inconvenience of clinic or pharmacy schedules, barriers to care and changes of residence, and people with stable housing with monthly appointment reminder were more likely to complete the treatment compared to those without stable housing or monthly reminder.
Dawson et al. (2016) [34]	USA	Cross-sectional	TB incidence of NYCHA residents was twice compared to that of non-NYCHA residents, and high TB strain diversity was found among NYCHA residents.
Khan et al. (2016) [44]	Canada	Case-control	The number of people per room was positively associated with the probability of newly diagnosed infection and disease, but only limited to the participants who lived with the one with smear-positive TB.
Yamin et al. (2016) [25]	USA	Cohort	The identified predictors of LTBI treatment incompletion were persons with unstable housing, tobacco use and those experiencing an adverse drug event.
Pedro et al. (2017) [45]	Brazil	Ecological	The housing conditions such as walls, sewage infrastructure and population density in the house was significantly associated with the incidence of TB.
Kerr et al. (2020) [33]	USA	Cross-sectional	The factors associated with recent TB evaluation were whether PEH was sheltered and PEH had awareness on the TB outbreak in the homeless.
**Africa**			
Cramm et al. (2011) [46]	South Africa	Cross-sectional	The households with overcrowding and roof leakage were more likely to experience a household TB, while such probability was significantly reduced with higher social capital.
Ephrem et al. (2015) [47]	Ethiopia	Case-control	The factors associated with active PTB were number of households in the compound and the number of windows in a house.
Tesema et al. (2015) [48]	Ethiopia	Case-control	The independently associated factors of TB development were illiteracy, households with more than four family members, room space less than 4 m^2^, non-separated kitchen, history of contact with a TB patient, a house with no ceiling and absence of windows.
Shimeles et al. (2019) [49]	Ethiopia	Case-control	Less number of windows was significantly related to increased TB risks.
Biru et al. (2020) [50]	Ethiopia	Case-control	DR-TB among TB patients were significantly influenced by the risk factors of living in a one-roomed house, history of contact with DR-TB cases, treatment failure TB cases and relapsed TB cases.
**Europe**			
Arnold et al. (2017) [29]	the UK	Cohort	The length of hospital admission was significantly associated with pulmonary TB, cavities on chest radiograph, a public health policy of waiting for sputum culture conversion and loss of patient’s home.

Note: The table is sorted in order of the continent by the number of studies, the published year within the continent, and alphabetical order of the first author’s name of each study

*TB* Tuberculosis, *LTBI* Latent Tuberculosis Infection, *COM* Community based intervention group, *FAC* TB Facility care group, *TAU* Treated As Usual group, *PEH* Person Experiencing Homelessness, *NYCHA* New York City Housing Authority, *DR-TB* Drug-Resistant Tuberculosis.

The effects of inadequate housing on TB were compared with those of adequate housing, and the significant differences in point estimate values were summarized (Table 2; see Additional file 2 for details). The exposure factors related to housing were categorized into housing affordability and quality. The consequent outcome factors that were quantified regarding the eight steps in TB development and the consequences thereof were summarized. Incidence, treatment adherence, and treatment success were included in the steps, and the incidence outcome included TB history, prevalence, and incidence rate.

**Table 2:** The association between inadequate housing and tuberculosis by continent

			Outcomes				
			TB history	Prevalence	Incidence Rate	Treatment non-adherence	Treatment success
**Exposures**	**Poor housing affordability**	**Homelessness**	**Homeless vs. Non-homeless(sheltered)**				
				++~+++ [36]		++ [28]	++ [28]
				+++ [34]			+ [37]
				++ [29]			+++ [32]
			**History of homelessness**				
					+ [35]		
		**Instability**		+++ [47]			++ [27]
				+ [14]			++^a^ [31]
				++^b^ [39]			+ [30]
				++^c^ [47]			
	**Poor housing quality**	**Overcrowding**	**Household with more than 2 residents per room**				
			++~+++ [42]	+++ [48]	+ [45]	++ [25]	
			+ [46]	+++ [13]	+ [44]		
				+ [47]			
				+++ [40]			
		**Quality of housing**	**Improper ventilation**				
			+++ [42]	+++ [43]			
				++ [49]			
				+++ [47]			
				+++ [48]			
			**Lack of sunlight**				
				+++ [43]			
			**Poor roof condition**				
			+ [46]	+ [48]			
				++ [47]			
			**Poor floor condition**				
				++ [47]			
			**Poor wall condition (without coating)**				
					+ [45]		
			**Housing material (kaccha/kacha vs. pucca/pacca)** ^d^				
			+++ [42]	++ [41]			

OR or RR 1<+≤2, 2<++≤3, 3<+++. TB, Tuberculosis. ^a^Prolonged hospitalization. ^b^Public vs. Private housing. ^c^Shared house. ^d^See text for details

In terms of housing affordability, TB prevalence, treatment non-adherence, and treatment failure were significantly higher among the homeless, defined as a resident of inadequate housing, than in the non-homeless. Also, TB prevalence was approximately twice as high in public or shared houses with housing instability than in private houses. Instability was defined as no housing security, and this category included public rental housing or a subsidized flat [38] and no ownership of any kind of housing [47].

All studies defined living with more than two people per room as “overcrowding”, with only one study using a mean of 1.5 persons as the cutoff [34]. Most studies did not mention room size, but one study used a cutoff of 4 m^2^ [48]. The criteria for poor ventilation, including the presence of windows [47], number of windows [48, 49], and “ventilation area of the house” [43] without a clear definition of the area, varied among studies, and the results for poorly ventilated areas were compared with those for non-poorly ventilated areas within the same study. Poor roof or floor conditions included leaking roofs [46], thatched roofs instead of corrugated iron sheets [47], and earth or other floors instead of cement [47]. In India and Pakistan, there is a type of housing called “*kaccha*” or “*kacha*”, which is a house made of straw and mud, compared with a brick house, called “*pucca*” or “*pacca*”. Those living in *kaccha* housing had more than a threefold higher rate of a TB history and over twofold higher TB prevalence compared with those living in *pucca* housing. The results of all included studies showed that TB history, prevalence, incidence rate, treatment non-adherence, and treatment success were all significantly higher in residents of inadequate housing compared to those living in adequate housing.

**Fig. 2:**
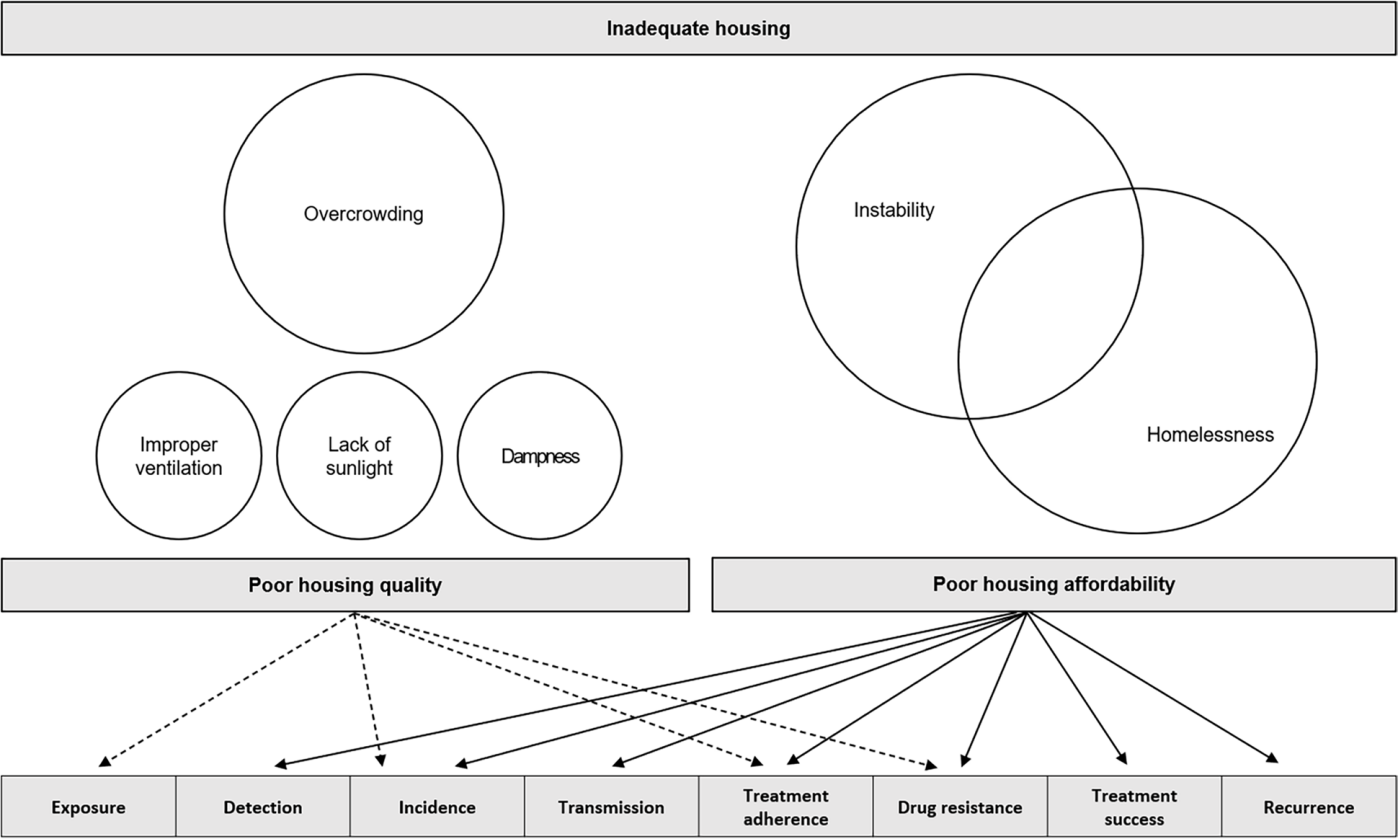
Inadequate housing factors, steps in TB development, and consequences

The included studies highlighted the problems associated with unaffordability of housing or the quality of poor housing and explain TB development and the consequences thereof. Based on existing studies, we classified TB development and its consequences into the following eight steps from the perspective of TB epidemiology: exposure, detection, incidence, transmission, treatment adherence, drug resistance, treatment success, and recurrence. The comprehensive results of the included studies represent the pathways that lead to the steps in TB development and the consequences thereof in terms of housing characteristics (Fig. 2).

The complete analysis showed that housing affordability was involved in more steps in TB development and its consequences than was housing quality. Housing affordability was identified by homelessness and instability, which are not separate entities. For example, because they are often reversible, homelessness and instability were considered to overlap, in that a person can become homeless after having to leave accommodation because of their inability to afford housing costs or vice versa. Housing quality was related to TB development and its consequences in terms of overcrowding, ventilation, sunlight, and dampness; dampness depends on the state of the roof, floor, and walls. The size of each circle expressed in Fig. 2 reflects the size of the effect. Specifically, compared with the other three factors, overcrowding had the greatest effect on TB outcomes. The arrows in the figure represent the pathways from the quality (dotted line) and affordability (solid line) of housing to each step in TB development and the consequences thereof. Specifically, housing affordability was linked to all steps, from detection to treatment success. The steps identified in TB development and the consequences thereof had a stronger association with housing affordability than with housing quality.

## Discussion

We systematically searched the English-language literature for studies of the effects of inadequate housing on the development of TB, and the consequences thereof. The study summarized the effects of inadequate housing, in terms of affordability and quality, on infection with TB. We reviewed 26 studies published since 2011, identified by a search of four reliable electronic databases. All eligible studies emphasized the importance of housing affordability and quality in the prevention, early detection, treatment, and management of TB. This study, which defined inadequate housing as unaffordable housing of low quality, confirmed that compared with residents of adequate housing, residents of inadequate housing had a significantly higher risk of developing each of the eight steps in TB.

We found that both the affordability and quality aspects of inadequate housing were associated with TB exposure (TB bacilli inhalation), incidence, and treatment adherence. We also found that poor housing affordability was associated with almost all eight steps of TB development and its consequences. Homelessness, which was highly related to TB infection, is increasing worldwide. The risk of further TB spread and the development of drug-resistant TB are serious issues, particularly in terms of the accelerated rate of homelessness in low-income countries [51], where the numbers of new or recurrent cases are high.

Poor housing quality indicates that the indoor environment is inadequate, although there is no problem with housing affordability. Overcrowding, such as shared housing, is a typical risk factor for TB [2, 7, 8, 52]. Overcrowding directly affects TB exposure, transmission, incidence, and drug resistance, and is also associated with lack of sunlight, dampness, and improper ventilation. The WHO defines crowding as a lack of space within a “dwelling for living, sleeping, and normal family/household life”. It has found a dose-response relationship between housing and health outcomes. Crowding measures the relationship between the number of residents in a space and the amount of residential space (i.e., rooms or floor area) available [53]. Studies that measure overcrowding should report the area of the living space and the number of persons per room instead of using arbitrary definitions.

Poor ventilation itself increases the risk of death regardless of socioeconomic status [54]. Along with crowding, poor ventilation has been identified as having negative effects on health; ventilation regulations differ from country to country and by the space in the house and are not easy to quantify [55]. Assessing ventilation with the presence of a window alone is inappropriate. Even if there is an operable window, it may not provide proper ventilation because of “outdoor temperature, noise, comfort, energy costs, the condition of windows or doors, or cultural and personal habits” [56]. Improper ventilation, resulting from no windows, *kaccha* housing, living on the lower floors of high-rise buildings, and overcrowding, is associated with disease transmission and increases the incidence of TB by prolonging the time that TB bacilli droplets remain suspended in indoor air. The lower floors of high-rise buildings (most public housing) can lead to a higher incidence of TB due to a lack of sunlight and improper ventilation. Uncoated walls or leaking roofs increase dampness in housing and are associated with an increased incidence of TB.

The most important urgent medical intervention for TB is to begin treating active TB before transmission. Drug resistance and treatment completion are major challenges in TB eradication; the latter can be hindered by multiple factors and poses a high risk of resulting in drug-resistant TB. The main reason TB patients stop treatment is related to their economic conditions, not only their income but also the cost of completing treatment [15], including transportation to the treatment facility, food for essential nutrition during treatment, and housing. Housing affordability due to reduced income during TB treatment can be both a reason for, and a result of, not being able to adhere to treatment. Poor housing affordability decreases rates of TB detection [33] and treatment adherence [26, 28] and access [25, 26, 28–30, 32]. In particular, homelessness increases the risk of the recurrence of TB [31]. The WHO has identified seven intervention categories for adherence to TB treatment: “supervising treatment (e.g., directly observed therapy), reminders and traces, incentives and enablers, patient education, digital technologies, staff education, and combinations of these interventions” [57]. Housing is directly or indirectly related to all intervention categories affecting adherence to TB treatment.

The importance of housing affordability has been confirmed by intervention studies of housing support, particularly as regards homelessness, resulting in reduced hospital stays and follow-up times for TB patients [27, 29, 30, 32]. Public health interventions may differ according to the step of disease development, but TB is a complex set of processes, and thus a combined intervention approach is important. Specifically, patient education is recommended to reduce the risk of TB infection [36], and subsidies [58] or treatment methods requiring a shorter period [26] all help to increase the success rate of treatment. Integrated preventive interventions for community health workers and professional TB centers can reduce delays in TB treatment and expand access to TB treatment facilities [42].

There are many risk factors associated with TB other than housing. These risk factors were included in the final analysis along with housing and were controlled. None of the 26 eligible studies tested the interaction between housing and other factors to verify the role of effect modifiers. The housing factor and TB were deemed to be independent in the selected studies. In the final model of each paper, the factors with the greatest effects other than housing were smoking and substance use. The risk factors from the selected studies are detailed in a separate table (see Additional file 4: Table S4_2).

The study distribution and housing issues of interest varied depending on the economic level of the country. High-income countries may recognize that TB is more prevalent with certain types of housing, such as homelessness [26, 27, 30–33, 35] or public [34] and shared [39] housing. It may be difficult for low- and middle-income countries to perceive housing as a risk factor of TB, as the prevalence of TB in the general population is still high. According to a WHO report, the prevalence of TB is still 150–400 cases per 100,000 population in 30 countries with high burden, and more than 500 cases per 100,000 population in the Central African Republic, the Democratic People’s Republic of Korea, Lesotho, the Philippines, and South Africa [1]. Furthermore, high-income countries may have a relatively sufficient budget and infrastructure to implement active strategies to end TB whereas in low- and middle-income countries, despite an increasing consensus that action to address social determinants of TB is necessary, practical ideas for such actions are scarce [4].

### Strengths and limitations

This systematic review was a rigorous review of studies of various designs conducted in different countries. However, there are several potential limitations to this work. First, we may have missed some important articles, as we only included literature published between Jan 1, 2011 and Oct 25, 2020. Second, due to the linguistic limitations of the researchers, the articles were limited to English only. Third, a meta-analysis could not be performed because of the heterogeneity among populations and studies. For example, some studies defined homelessness as street homeless only, without specific explanation, while others defined homelessness as living on the streets, in shelters, or in health facilities. There were some differences in the definition of treatment completion or success, such as when more than 88% of the prescribed regimen was achieved or when a negative smear was confirmed after 4 months of treatment.

Nevertheless, this is the first systematic review to evaluate the effects of inadequate housing on TB and to categorize inadequate housing in terms of affordability and quality. We attempted to define the pathways leading to TB in terms of housing characteristics based on housing and health frameworks [13, 19] and frameworks of proximal risk factors for TB [7, 8]. We found that housing of inadequate quality increased the risk of TB development due to TB exposure and transmission, rendering it difficult to adhere to treatment and increasing the risk of multidrug-resistant TB. Without affordable housing, detection of TB infection, exposure and incidence, and of successful treatment completion, which affect all eight steps of TB detection, treatment and prevention are difficult.

## Conclusions

Consequently, we propose that housing affordability, together with housing quality, should be considered a proximal risk factor for TB, and that housing affordability is associated with more steps in TB development and its consequences than is housing quality. It is necessary to combine and apply evidence-based public health interventions at each step in the process of TB development and the consequences thereof. Interventions regarding housing affordability are likely to be diverse. Further research on the associations between the incidence and treatment of TB and long-term housing stability and between the assessment of the incidence of TB and housing interventions that might have significant synergistic effects with other interventions could provide a strong scientific basis for establishing more effective TB-prevention policies.

## Supplementary Information

**Additional file 1: Table S1.** Characteristics of selected studies on tuberculosis and inadequate housing.

**Additional file 2: Table S2.** Comparative results for the target population by characteristics of inadequate housing.

**Additional file 3: Table S3.** Studies excluded at the full-text screening stage, with brief explanations.

**Additional file 4: Table S4_1.** Results for risk of bias assessed using the JBI checklists. **Table S4_2.** Risk factors for TB other than housing.

**Additional file 5: Appendix 1.** PRISMA Checklist. **Appendix 2.** Review protocol.

## Data Availability

Data sharing is not applicable to this article as no datasets were generated or analysed during the current study.
