# Supplementary material for: Inadequate housing and pulmonary tuberculosis: a systematic review
Source: BMC Public Health. 2022 Mar 30;22:622. doi: 10.1186/s12889-022-12879-6 (PMC8966856; doi:10.1186/s12889-022-12879-6)
Supplement: Supplementary file 1 — Additional file 1: Table S1. Characteristics of selected studies on tuberculosis and inadequate housing. [file 12889_2022_12879_MOESM1_ESM.docx]

**Table S1. Characteristics of selected studies on tuberculosis and inadequate housing**

| **continent** | **1st author** | **Country, region** | **Period** | **Data source** | **Exposure variable** | **Steps of TB** | **Definitions** |
| --- | --- | --- | --- | --- | --- | --- | --- |
| Asia | Heo et al. (2012) [14] | South Korea, Busan | 2001-2010 | Busan medical center data | Homelessness (homeless vs. non-homeless (sheltered)) | Treatment success | 1) Cured: a patient who is smear-negative after the treatment completion (in the last month of the treatment) and on at least one previous occasion.  2) Treatment failure: a smear-positive patient after four months of treatment or whose DST shows MDR-TB. 3) Treatment default: a patient whose treatment was interrupted before the completion of the pre-arranged treatment by the doctor, including one who was lost during the follow-up after his/her discharge or who escaped from the hospital. |
|  | Haider et al. (2013) [24] | Pakistan, Karachi | 2003 | Patient's data extracted from tertiary hospital | Housing Instability | Exposure | 1) Overcrowding: more than 2 persons per habitable room. 2) Habitable rooms: all rooms of the house excluding kitchen, storeroom, verandah and bathrooms. |
|  | Lai et al. (2013) [25] | Hong Kong, Kowloon | 2007-2009 | The TB and Chest Services of hospitals | Quality of housing | Incidence | Natural daylight capacity: the Sky View Factor which is a measure of the openness of the sky relative to a specific location with values ranging from 0 (no sky visible) to 1 (no foliage/obstruction visible) |
|  | Lee et al. (2013) [16] | South Korea, Seoul | 2009-2010 | 5 homeless shelters in Seoul | Homelessness (homeless vs. non-homeless (sheltered)) | TB Recurrence |  |
|  | Low et al. (2013) [26] | Hong Kong | 2007-2009 | Centralized Mycobacterium Laboratory | Housing Instability | Incidence |  |
|  | Choi, H. et al.(2016) [20] | South Korea | 2005-2012 | Clinical cohort data from 2 hospitals | Overcrowding (shared house) | Treatment adherence | Poor adherence: treatment interruption for at least 2 consecutive months and not restarting the same regimen within 6 months |
|  | Irfan et al. (2017) [27] | Bangladesh | N/A | Face-to-face interview | Overcrowding | Incidence | Overcrowding: Household with more than 2 residents per room, family members to room ratio (≤2, 2+) |
|  | Rao et al. (2018) [21] | India, Madhya Pradesh | 2013-2014 | house-to-house survey | Quality of housing | Incidence |  |
|  | Kim et al. (2019) [19] | South Korea, Seoul | 2016-2018 | Clinical charts from 3 hospitals in Seoul | Homelessness (homeless vs. non-homeless (sheltered)) | Treatment success | Homelessness: the state of living without a shelter or living quarters (primary homelessness), including a no place of usual residence (secondary homelessness), or lacking in access to minimally adequate housing. |
|  | Saqib et al. (2019) [23] | Pakistan | 2016-2017 | Interview & Survey | Overcrowding & Quality of housing | Transmission | Overcrowding: number of people in the household and number of rooms in the home. |
|  | Wardani et al. (2019) [22] | Indonesia | 2016 | Sukaraja and Panjang Community Health Centre | Quality of housing | Incidence | 1) Ventilation measure: percentage of ventilation area of house width (less ventilation: <20%, adequate ventilation: ≥20%) 2) In-house sunlight: observed by existence of sunlight in-house |
| America | Kerker et al. (2011) [18] | USA | 2001-2003 | The New York City Bureau of Vital Statistics | Homelessness (homeless vs. non-homeless (sheltered)) | Transmission | Housing Assistance: the Department of Homeless Services supplies apartment-style shelters and support services such as childcare, housing assistance, and health care referral to homeless families. |
|  | Bamrah et al. (2013) [10] | USA | 2006-2010 | TB cases reported to the National Tuberculosis Surveillance System, the US Department of Housing and Urban Development homeless population estimates | Homelessness (homeless vs. non-homeless (sheltered)) | Treatment success | 1) Homeless with TB: if during the 12 months before the initial diagnostic evaluation for TB, the person lacked a fixed, regular and adequate night-time residence; had a primary night-time residence that was a supervised publicly or privately operated shelter, an institution that provides temporary residence, or a public or private building not designated for, or ordinarily used as, a regular sleeping accommodation for human beings; or had no home or was alternating between multiple residences. 2) TB genotype cluster: ≥2 TB cases in the same county during 2004–2010 with matching spoligotype and 12-locus mycobacterial interspersed repetitive unit–variable number tandem repeats. |
|  | Feske et al. (2013) [11] | USA | 1995-2004 | Surveillance data | Homelessness (homeless vs. non-homeless (sheltered)) | Treatment coverage |  |
|  | Hirsch-Moverman et al. (2015) [12] | USA & Canada | 2007-2008 | medical charts and clinic surveys | Housing Instability | Treatment adherence | Treatment completion: specified number of doses completed within a specified time period for each regimen. |
|  | Dawson et al. (2016) [17] | USA | 2001-2009 | New York City TB surveillance registry | Homelessness (history of homelessness) | Incidence | Severe overcrowding: proportion of units with mean persons per room more than 1.5 in one’s neighborhood. |
|  | Khan et al. (2016) [29] | Canada | 2011-2012 | the contacts and from the study questionnaires | Overcrowding | Incidence | Overcrowding: Household with more than 2 residents per room, more than 1 person per room in the dwelling |
|  | Yamin et al. (2016) [9] | USA | 2012-2013 | Clinical chart extracted from the Fulton County Health Department TB clinic | Housing Instability | Treatment success | 1) Treatment completion: finishing ≥88% of the prescribed regimen. 2) Interruption of treatment: missing ≥7 days of medication for any regimen. |
|  | Pedro et al. (2017) [28] | Brazil | 2006-201 | Sistema Nacional de Agravos de Notificação | Overcrowding | Incidence | Overcrowding: Household with more than 2 residents per room |
|  | Kerr et al. (2020) [15] | USA | 2015-2016 | Atlanta Continuum of Care | Homelessness (history of homelessness) | Transmission, Detection & Treatment coverage | 1) Sheltered PEH: persons living in a supervised, publicly or privately-operated shelter designated to provide a temporary living arrangement on the night of the PIT count.  2) Unsheltered PEH: persons staying in public or private places not designed for or ordinarily used as regular sleeping accommodations (eg, under a bridge, in a tent) during the period between dusk and dawn on the night designated for the PIT count. |
| Africa | Cramm et al. (2011) [34] | South Africa, Grahamstown East/Rhini | 2007 | Survey of 1020 Grahamstown East/Rhini households | Overcrowding & Quality of Housing | Incidence | 1) Overcrowding: Household with more than 2 residents per room  2) Poor roof condition: leaking roof |
|  | Ephrem et al. (2015) [33] | Ethiopia | 2011-2012 | Pre-tested structured questionnaire | Housing Instability, Overcrowding & Quality of housing | Transmission | 1) Housing instability: ownership of the house (yes, no) 2) Overcrowding: Household with more than 2 residents per room, persons per room (≤2, >2) 3) Poor ventilation: existence of windows (present, absent) 4) Poor roof condition: type of roof (corrugated iron sheet, Thatch) 5) Poor floor condition: type of floor (cement, earth/other) |
|  | Tesema et al. (2015) [31] | Ethiopia | 2013 | Face-to-face interview | Overcrowding & Quality of housing | Incidence | 1) Overcrowding: Household with more than 2 residents per room, area of the room per person was less than 4m^2^ 2) Poor ventilation: number of windows (0, 1, >1) |
|  | Shimeles et al. (2019) [30] | Ethiopia, Addis Ababa | 2017 | 20 health centers in Addis Ababa | Quality of housing | Incidence | Poor ventilation: number of windows (0-1, 2-3, >3) |
|  | Biru et al. (2020) [32] | Ethiopia | 2016-2017 | survey | Overcrowding | Drug-resistance | Shared house: number of rooms in the house (1, ≥2) |
| Europe | Arnold et al. (2017) [13] | the UK | 2008-2014 | Clinical records and hospital database review at 4 TB treatment centers | Housing Instability | Treatment coverage & Drug-resistance | 1) Sputum CC from positive: the date of the first of two samples cultured to negativity taken at least 30 days apart, or culture negativity on one sample with no further samples and no positive samples in the 7 days prior.  2) Time to CC: the time between MDR-TB drug initiation and CC.  3) Culture reversion to positive: one sample that was culture positive taken over 30 days from the date of initial sputum CC or one positive sputum after the end of the injectable if CC not documented. |

Note: The table is sorted in order of the continent by the number of studies, the published year within the continent, and alphabetical order of the first author's name of each study.

TB, Tuberculosis; DST, Drug Susceptibility Test; MDR-TB, Multidrug-Resistant Tuberculosis; PEH, Person Experiencing Homelessness; PIT, Point-In-Time; CC, Culture Conversion.
