# Supplementary material for: Inadequate housing and pulmonary tuberculosis: a systematic review
Source: BMC Public Health. 2022 Mar 30;22:622. doi: 10.1186/s12889-022-12879-6 (PMC8966856; doi:10.1186/s12889-022-12879-6)
Supplement: Supplementary file 2 — Additional file 2: Table S2. Comparative results for the target population by characteristics of inadequate housing. [file 12889_2022_12879_MOESM2_ESM.docx]

**Table S2. Comparative results for the target population by characteristics of inadequate housing**

|  | **Target population characteristics(n)**  **(Comparator(n))** | **Outcome** | **Range among target population** | |
| --- | --- | --- | --- | --- |
|  |  |  | **Value** | **95% CI** |
| **Poor housing affordability** | | | | |
| Kerker et al. (2011) [18] | TB cases who used the DHS family shelters (n=14)  (TB cases among NYC Total Population (n=3280),  TB cases among NYC Low-Income Neighborhoods (n=241)) | Prevalence | RR 3.6 (vs. TB cases among NYC total population) | 2.2-6.0 |
|  |  |  | RR 2.7 (vs. TB cases among NYC low-income neighborhoods) | 1.6-4.5 |
| Heo et al. (2012) [14] | Street homeless (n=65)  (Homeless in shelter or health facility (n=65)) | Poor treatment completement | aOR 4.8 | 2.1-11.1 |
| Bamrah et al. (2013) [10] | Homeless (n=16527)  (Non-homeless (n=254421)) | Completed treatment | aOR 0.4 | 0.4-0.5 |
|  |  | Moved, lost or refused treatment | aOR 2.6 | 2.4-2.9 |
| Feske et al. (2013) [11] | Transiently Housed (n=83),  Living in Shelter (n=114),  Living on the Street (n=248)  (Housed (n=2899)) | Prevalence | RRR 2.4 (living on the street) | 1.1-5.6 |
|  |  | Hospitalized days | >70 days (vs. 13.6 days) |  |
| Haider et al. (2013) [24] | Rented house (n=129 out of 342)  (Owned house (n=97 out of 342)) | Prevalence | aOR 1.6 | 1.1-2.3 |
| Lee et al. (2013) [16] | Homeless (n=289)  (Non-homeless (n=1796)) | LTBI prevalence | aPRR 24.9 | 14.2-40.4 |
| Hirsch-Moverman et al. (2015) [12] | Moved frequently (n=306)  (Stable housing (n=632)) | Treatment adherence | aRR 0.6 | 0.4-0.8 |
| Choi, H. et al. (2016) [20] | Shared housing (n=38)  (Private housing (n=513)) | Poor treatment adherence | aOR 2.6 | 1.2-5.6 |
| Dawson et al. (2016) [17] | TB cases with homeless history (n=512)  (TB cases without homeless history (n=8441)) | Incidence rate | OR 1.3 | 1.0-1.7 |
| Yamin et al. (2016) [9] | Unstable housing (n=135)  (Stable housing (n=289)) | Completing LTBI treatment | aOR 0.4 | 0.2-0.7 |
| Arnold et al. (2017) [13] | Loss of home while an inpatient for those admitted (n=10)  (Housed patient (n=55)) | Duration of hospital admission | aOR 1.8 | 1.2-2.7 |
| Kim et al. (2019) [19] | COM (n=50) and FAC (n=153)  (TAU (n=115)) | Treatment success rate | aOR 4.2 (COM) | 1.6-10.8 |
|  |  |  | aOR 17.0 (FAC) | 6.8-42.8 |
| Kerr et al. (2020) [15] | Sheltered PEH (n=741)  (Unsheltered PEH (n=628)) | TB evaluation | aOR 2.1 | 1.4-3.1 |
|  |  | Awareness of the TB outbreak among the homeless | aOR 3.3 | 2.3-4.8 |
| **Poor housing quality** | | | | |
| Cramm et al. (2011) [34] | TB history case within the HH (n=329)  (No TB history case within the HH (n=691)) | TB history | aOR 1.2 (overcrowding) | 1.0-1.3 |
|  |  |  | aOR 1.2 (leaking roof) | 1.0-1.4 |
| Lai et al. (2013) [25] | TB cases (n=1227) | Prevalence | Interaction between QUINTILE (floor)*BUILDING (tall/high rise): statistically significant | |
| Low et al. (2013) [26] | Public housing (n=767)  (Private housing (n=355)) | Prevalence per 100,000 | 33.31 (vs. 15.42) | |
| Ephrem et al. (2015) [33] | PTB cases (n=104)  (Non-PTB cases (n=208)) | Prevalence | OR 2.3 (the number of HHs in the compound (>1)) | 1.4-4.0 |
|  |  |  | OR 1.6 (the number of persons per room (>2)) | 0.9-2.7 |
|  |  |  | OR 2.2 (type of house floor (earth)) | 1.0-4.8 |
|  |  |  | OR 2.4 (type of house roof (thatch)) | 1.2-4.6 |
|  |  |  | OR 3.0 (no window) | 1.6-5.7 |
|  |  |  | OR 3.7 (no ownership of the house) | 2.1-6.5 |
| Tesema et al. (2015) [31] | PTB cases (n=218)  (Non-PTB cases (n=437)) | Prevalence | aOR 3.1 (HH containing more than four family members) | 2.1-4.6 |
|  |  |  | aOR 4.4 (absence of windows) | 2.5-8.0 |
| Khan et al. (2016) [29] | 1.7 people per room (n=88)  (1.1 people per room (n=67)) | Incidence | aOR 1.8 (among participants living with smear-positive person) | 1.1-2.9 |
| Irfan et al. (2017) [27] | TB cases (n=178)  (Non-TB cases (n=179)) | Prevalence | OR 3.5 (overcrowding in a house (>2 HH per room)) | 2.1-5.9 |
| Pedro et al. (2017) [28] | PTB cases (n=804) | Incidence rate | IRR 1.8 (HH without coating on the external walls) | 1.6-2.1 |
|  |  |  | IRR 1.4 (HH with more than two residents per room) | 1.1-1.8 |
| Rao et al. (2018) [21] | Living in Kaccha house type (n=201 out of 220)  (Living in Pucca house type (n=634 out of 660)) | Prevalence | aOR 2.7 | 1.4-5.1 |
| Saqib et al. (2019) [23] | TB history cases (n=165)  (No TB history cases (n=104)) | TB history | aOR 14.9 (Kacha) | 3.8-57.6 |
|  |  |  | aOR 0.4 (3-4 Rooms in house) | 0.1-1.8 |
|  |  |  | aOR 0.0 (>4 Rooms in house) | 0.00-0.19 |
|  |  |  | aOR 0.2 (good ventilation) | 0.1-0.6 |
| Shimeles et al. (2019) [30] | Participants with no or 1 window (n=181 out of 260)  (Participants with multiple windows (n=145 out of 260)) | Prevalence | aOR 1.8 | 1.1-3.1 |
| Wardani et al. (2019) [22] | TB cases (n=31)  (Non-TB cases (n=62)) | Less ventilation | OR 4.7 | 1.9-12.0 |
|  |  | No in-house sunlight | OR 5.2 | 2.0-13.4 |
| Biru et al. (2020) [32] | DR-TB cases (n=84)  (Non-DR-TB cases (n=243)) | Prevalence | aOR 6.8 (living in a one-roomed house) | 1.8-25.8 |

Note: Statistically significant values are presented only. All values are rounded up to one decimal point. The study results were sorted according to the order of the published year.

OR, Odds Ratio; aOR, adjusted Odds Ratio; aRR, adjusted Relative Risk; CI, Confidence Interval; RRR, Relative Risk Ratio; aPRR, adjusted Prevalence Rate Ratio; IRR, Incidence Rate Ratio; LTBI, Latent Tuberculosis Infection; PEH, Person Experiencing Homelessness; TB, Tuberculosis; PTB, Pulmonary Tuberculosis; DR-TB, Drug-Resistant Tuberculosis; TAU, Treated As Usual group; COM, Community-based intervention group; FAC, TB Facility Care group; DHS, Department of Homeless Services; NYC, New York City; HH, Household.
