# Supplementary material for: Inadequate housing and pulmonary tuberculosis: a systematic review
Source: BMC Public Health. 2022 Mar 30;22:622. doi: 10.1186/s12889-022-12879-6 (PMC8966856; doi:10.1186/s12889-022-12879-6)
Supplement: Supplementary file 3 — Additional file 3: Table S3. Studies excluded at the full-text screening stage, with brief explanations. [file 12889_2022_12879_MOESM3_ESM.docx]

**Table S3. Studies excluded at the full-text screening stage, with brief explanations**

| no. | exclusion criteria |
| --- | --- |
| 1 | housing not explained in detail |
| 2 | not related to pulmonary tuberculosis |
| 3 | could not obtain full text |
| 4 | not related to steps of TB development and its consequences |
| 5 | not written in English |
| 6 | study done before the year 2000 |
| 7 | clinical study or case report |
| 8 | duplicate |
| 9 | encyclopedia or glossary entry |
| 10 | did not involve human participants |

| citation | Criteria number |
| --- | --- |
| Ajayi, I. O., Jegede, A. S., Falade, C. O., & Sommerfeld, J. (2013). Assessing resources for implementing a community directed intervention (CDI) strategy in delivering multiple health interventions in urban poor communities in Southwestern Nigeria: a qualitative study. Infectious Diseases of Poverty, 2. doi:10.1186/2049-9957-2-25 | 2 |
| Aguirre, S., Cuellar, C. M., Herrero, M. B., Cortesi, G. C., de Romero, N. G., Alvarez, M., & Braga, J. U. (2017). Prevalence of tuberculosis respiratory symptoms and associated factors in the indigenous populations of Paraguay (2012). Memorias Do Instituto Oswaldo Cruz, 112(7), 474-484. doi:10.1590/0074-02760160443 | 2 |
| Alcaide, F., & Coll, P. (2011). Advances in rapid diagnosis of tuberculosis disease and anti-tuberculous drug resistance. Enfermedades Infecciosas Y Microbiologia Clinica, 29, 34-40. doi:10.1016/s0213-005x(11)70016-7 | 4 |
| Ankale, P., Nair, G., Uppe, A., Mathew, A., & Shah, R. (2017). Socioeconomic conditions contributing to multi drug resistant (MDR) and Extremely Drug Resistant(XDR) Tuberculosis. European Respiratory Journal, 50. doi:10.1183/1393003.congress-2017.PA2727 | 3 |
| Arbore, A. S., & Cojocariu, V. (2019). Homeless with TB: A “long race” vulnerable group (20 years review). European Respiratory Journal, 54. doi:10.1183/13993003.congress-2019.PA5273 | 3 |
| Arianna, A. H. R., MaríaTeresa, E. M., & Carlos, R. L. J. (2014). Risk of becoming Ill with pulmonary tuberculosis in Mexico. Biosciences Biotechnology Research Asia, 11(2), 773-777. doi:10.13005/bbra/1335 | 1 |
| Arshad, M., Ifrahim, M., Ashraf, M., Rehman, S. U., & Khan, H. A. (2012). EPIDEMIOLOGICAL STUDIES ON TUBERCULOSIS IN BUFFALO POPULATION IN VILLAGES AROUND FAISALABAD. Journal of Animal and Plant Sciences, 22, 246-249. Retrieved from <Go to ISI>://WOS:000209472600025 | 10 |
| Asefa, A., & Teshome, W. (2014). Total Delay in Treatment among Smear Positive Pulmonary Tuberculosis Patients in Five Primary Health Centers, Southern Ethiopia: A Cross Sectional Study. PLoS One, 9(7). doi:10.1371/journal.pone.0102884 | 1 |
| Asyary, A., Eryando, T., Purwantyastuti, Junadi, P., Clark, C., & van Teijlingen, E. (2017). Level of Exposure to Childhood Tuberculosis in Household Contacts with Adult Pulmonary Tuberculosis. Kesmas-National Public Health Journal, 12(1), 1-6. doi:10.21109/kesmas.v12i1.1469 | 1 |
| Asyary, A., Junadi, P., Purwantyastuti, & Eryando, T. (2017). Socio-Economics of Childhood Pulmonary Tuberculosis with Adult Tuberculosis Household Contacts in Daerah Istimewa Yogyakarta Province. Makara Journal of Health Research, 21(3), 93-98. doi:10.7454/msk.v21i3.7550 | 1 |
| Azevedo, M. J., Conwill, D. E., Lawrence, S., Jackson, A., Bhuiyan, A. R., Hall, D., . . . Beckett, G. (2015). Tuberculosis Containment among the Homeless in Metropolitan Jackson, Mississippi. J Miss State Med Assoc, 56(8), 243-248. | 3 |
| Bae MH,Song BK,Kim KM,Son SK,Park SE. A Study on Clinical Manifestations of Pulmonary Tuberculosis and Tuberculosis Contact Investigation in School-Age Children and Adolescents at Two Centers. Korean J Pediatr Infect Dis. 2014 Dec;21(3):191-198. | 1 |
| Bamrah, S., Yelk Woodruff, R., Powell, K., & Haddad, M. (2012). Tuberculosis among persons experiencing homelessness-United States, 1994-2009. American Journal of Respiratory and Critical Care Medicine, 185. Retrieved from https://www.embase.com/search/results?subaction=viewrecord&id=L71986848&from=export | 3 |
| Baral, S. C., Aryal, Y., Bhattrai, R., King, R., & Newell, J. N. (2014). The importance of providing counselling and financial support to patients receiving treatment for multi-drug resistant TB: mixed method qualitative and pilot intervention studies. BMC Public Health, 14. doi:10.1186/1471-2458-14-46 | 1 |
| Beijer, U., Wolf, A., & Fazel, S. (2012). Prevalence of tuberculosis, hepatitis C virus, and HIV in homeless people: a systematic review and meta-analysis. Lancet Infectious Diseases, 12(11), 859-870. doi:10.1016/s1473-3099(12)70177-9 | 2 |
| Beyanga, M., Kidenya, B. R., Gerwing-Adima, L., Ochodo, E., Mshana, S. E., & Kasang, C. (2018). Investigation of household contacts of pulmonary tuberculosis patients increases case detection in Mwanza City, Tanzania. BMC Infectious Diseases, 18. doi:10.1186/s12879-018-3036-6 | 1 |
| Bhargava, A., Bhargava, M., & Juneja, A. (2020). Social determinants of tuberculosis: context, framework, and the way forward to ending TB in India. Expert Rev Respir Med, 1-17. doi:10.1080/17476348.2021.1832469 | 9 |
| Bhargava, A., Pai, M., Bhargava, M., Marais, B., & Menzies, D. (2012). Can Social Interventions Prevent Tuberculosis? The Papworth Experiment (1918-1943) Revisited. American Journal of Respiratory and Critical Care Medicine, 186(5), 442-449. doi:10.1164/rccm.201201-0023OC | 1 |
| Bhatt, G., Vyas, S., & Trivedi, K. (2012). An epidemiological study of Multi Drug Resistant Tuberculosis cases registered under revised national tuberculosis control programme of Ahmedabad City. Indian Journal of Tuberculosis, 59(1), 18-27. Retrieved from https://www.embase.com/search/results?subaction=viewrecord&id=L365267016&from=export | 3 |
| Bodeker, G. (2017). Health Care of Indigenous Peoples/Nations. In S. R. Quah (Ed.), International Encyclopedia of Public Health (Second Edition) (pp. 399-405). Oxford: Academic Press. | 3 |
| Bolongei, M. B., Jeuronlon, M. K., & Mulbah, G. J. (2019). Trends of tuberculosis and incidence of antibiotic resistance (Rifampicin) among tuberculosis cases: Implications for antimicrobial stewardship programming in Liberia. Antimicrobial Resistance and Infection Control, 8. doi:10.1186/s13756-019-0567-6 | 3 |
| Burrows NR, Geiss LS, Engelgau MM, Acton KJ. Prevalence of diabetes among Native Americans and Alaska Natives, 1990.1997: an increasing burden. Diabetes Care 2000; 23: 1786.90. | 6 |
| Calderon, M., Alvarado-Villacorta, R., Barrios, M., Quiroz-Robladillo, D., Naupay, D. R. G., Obregon, A., . . . Moore, D. (2019). Health need assessment in an indigenous high-altitude population living on an island in Lake Titicaca, Peru. International Journal for Equity in Health, 18. doi:10.1186/s12939-019-0993-3 | 2 |
| Cardoso, B. A., Fonseca, F. D., Neto, A. H. D., Martins, A., Oliveira, N. V. D., Lima, L., . . . Saad, M. H. F. (2017). Environmental aspects related to tuberculosis and intestinal parasites in a low-income community of the Brazilian Amazon. Revista do Instituto de Medicina Tropical de Sao Paulo, 59. doi:10.1590/s1678-9946201759057 | 1 |
| Carter J, Horowitz R, Wilson R, Sava S, Sinnock P, Gohdes D. Tribal differences in diabetes: prevalence among American Indians in New Mexico. Public Health Reports 1989; 104: 665.69. | 6 |
| Cavanaugh, J. S., Powell, K., Renwick, O. J., Davis, K. L., Hilliard, A., Benjamin, C., & Mitruka, K. (2012). An outbreak of tuberculosis among adults with mental illness. American Journal of Psychiatry, 169(6), 569-575. doi:10.1176/appi.ajp.2011.11081311 | 1 |
| Centers for Disease C, Prevention. Prevalence of diagnosed diabetes among American Indians/Alaskan Natives.United States, 1996. MMWR Morb Mortal Wkly Rep 1998; 47: 901.04. | 6 |
| Cha, O. (2013). [Health of the homeless]. Bull Acad Natl Med, 197(2), 277-289; discussion 289-291. doi:10.1016/s0001-4079(19)31584-5 | 5 |
| Chadha, V. K., Sarin, R., Narang, P., John, K. R., Chopra, K. K., Jitendra, R., . . . Kumar, P. (2013). Trends in the annual risk of tuberculous infection in India. International Journal of Tuberculosis and Lung Disease, 17(3), 312-319. doi:10.5588/ijtld.12.0330 | 1 |
| Choi SE, Liu M, Palaniappan LP, Wang EJ, Wong ND. Gender and ethnic differences in the prevalence of type 2 diabetes among Asian subgroups in California. J Diabetes Complications 2013; 27: 429.35. | 2 |
| Clark, M., Riben, P., & Nowgesic, E. (2002). The association of housing density, isolation and tuberculosis in Canadian First Nations communities. International journal of epidemiology, 31(5), 940-945. | 6 |
| Coimbra, C. E. A. (2014). Health and indigenous peoples in Brazil: reflections based on the First National Survey of Indigenous People's Health and Nutrition. Cadernos De Saude Publica, 30(4), 855-859. doi:10.1590/0102-311x00031214 | 5 |
| Condeng, B., Syafar, M., Sirajuddin, S., Naiem, F., & Saleh, A. (2020). Condition of houses with smoking habits in patients with pulmonary tuberculosis. Medico-Legal Update, 20(1), 245-249. doi:10.37506/v20/il/2020/mlu/194331 | 1 |
| Corbett, S., & Drisko, J. (2011). Health Conditions among the Potawatomi Indians of Kansas in 1928. Plains Anthropologist, 56(219), 215-242. doi:10.1179/pan.2011.018 | 6 |
| Cormier, M., Schwartzman, K., N'Diaye, D. S., Boone, C. E., Dos Santos, A. M., Gaspar, J., . . . Oxlade, O. (2019). Proximate determinants of tuberculosis in Indigenous peoples worldwide: a systematic review. Lancet Glob Health, 7(1), e68-e80. doi:10.1016/s2214-109x(18)30435-2 | 1 |
| Cramm, J. M., & Nieboer, A. P. (2011). The influence of social capital and socio-economic conditions on self-rated health among residents of an economically and health-deprived South African township. International Journal for Equity in Health, 10. doi:10.1186/1475-9276-10-51 | 2 |
| Dawson, P., Harris, T. G., Ahuja, S. D., Anderson, J. A., & Perri, B. R. (2012). Epidemiology of tuberculosis among New Yorkers living in public housing, 2001-2009. American Journal of Respiratory and Critical Care Medicine, 185. Retrieved from https://www.embase.com/search/results?subaction=viewrecord&id=L71986851&from=export | 3 |
| de Andrade, K. V. F., Nery, J. S., de Araujo, G. S., Barreto, M. L., & Pereira, S. M. (2019). Association between treatment outcome, sociodemographic characteristics and social benefits received by individuals with tuberculosis in Salvador, Bahia, Brazil, 2014-2016. Epidemiologia E Servicos De Saude, 28(2). doi:10.5123/s1679-49742019000200004 | 1 |
| de Oliveira, M. F., Arcencio, R. A., Ruffino-Netto, A., Scatena, L. M., Palha, P. F., & Villa, T. C. S. (2011). The front door of the Ribeirao Preto Health System for diagnosing tuberculosis. Revista Da Escola De Enfermagem Da Usp, 45(4), 893-898. Retrieved from <Go to ISI>://WOS:000294442100015 | 1 |
| de Oliveira, N. F., & Gonçalves, M. J. (2013). Social and environmental factors associated with the hospitalization of tuberculosis patients. Rev Lat Am Enfermagem, 21(2), 507-514. doi:10.1590/s0104-11692013000200006 | 2 |
| Dehghani, K., Lan, Z., Li, P., Michelsen, S. W., Waites, S., Benedetti, A., . . . Menzies, D. (2018). Determinants of tuberculosis trends in six Indigenous populations of the USA, Canada, and Greenland from 1960 to 2014: a population-based study. Lancet Public Health, 3(3), e133-e142. doi:10.1016/s2468-2667(18)30002-1 | 4 |
| Doherty, M., Power, L., Petrova, M., Gunn, S., Powell, R., Coghlan, R., . . . Khan, F. (2020). Illness-related suffering and need for palliative care in Rohingya refugees and caregivers in Bangladesh: A cross-sectional study. Plos Medicine, 17(3). doi:10.1371/journal.pmed.1003011 | 2 |
| Duarte, R., Santos, J. V., Silva, A. S., & Sotgiu, G. (2018). Epidemiology and socioeconomic determinants. ERS Monograph, 2018(9781849841009), 28-35. doi:10.1183/2312508X.10020717 | 1 |
| Ebbesson SO, Schraer CD, Risica PM, et al. Diabetes and impaired glucose tolerance in three Alaskan Eskimo populations. The Alaska-Siberia Project. Diabetes Care 1998; 21: 563.9. | 6 |
| Eldin, G. S. S., Fadl-Elmula, I., Ali, M. S., Ali, A. B., Salih, A., Mallard, K., . . . McNerney, R. (2011). Tuberculosis in Sudan: a study of Mycobacterium tuberculosis strain genotype and susceptibility to anti-tuberculosis drugs. BMC Infectious Diseases, 11. doi:10.1186/1471-2334-11-219 | 1 |
| Faust, L., McCarthy, A., & Schreiber, Y. (2018). Recommendations for the screening of paediatric latent tuberculosis infection in indigenous communities: a systematic review of screening strategies among high-risk groups in low-incidence countries. BMC Public Health, 18(1), 979. doi:10.1186/s12889-018-5886-7 | 1 |
| Firdaus, G., & Ahmad, A. (2013). Relationship between housing and health: A cross-sectional study of an urban centre of India. Indoor and Built Environment, 22(3), 498-507. doi:10.1177/1420326X12443846 | 2 |
| Friedman, E. E., Dean, H. D., & Duffus, W. A. (2018). Incorporation of Social Determinants of Health in the Peer-Reviewed Literature: A Systematic Review of Articles Authored by the National Center for HIV/AIDS, Viral Hepatitis, STD, and TB Prevention. Public Health Rep, 133(4), 392-412. doi:10.1177/0033354918774788 | 2 |
| Garcia-Basteiro, A. L., Hurtado, J. C., Castillo, P., Fernandes, F., Navarro, M., Lovane, L., . . . Martinez, M. J. (2019). Unmasking the hidden tuberculosis mortality burden in a large post mortem study in Maputo Central Hospital, Mozambique. European Respiratory Journal, 54(3). doi:10.1183/13993003.00312-2019 | 4 |
| Goetsch, U., Bellinger, O. K., Buettel, K. L., & Gottschalk, R. (2012). Tuberculosis among drug users and homeless persons: impact of voluntary X-ray investigation on active case finding. Infection, 40(4), 389-395. | 1 |
| Goldhaber-Fiebert, J. D., Jeon, C. Y., Cohen, T., & Murray, M. B. (2011). Diabetes mellitus and tuberculosis in countries with high tuberculosis burdens: individual risks and social determinants. Int J Epidemiol, 40(2), 417-428. doi:10.1093/ije/dyq238 | 1 |
| Gomes, V. F., Andersen, A., Wejse, C., Oliveira, I., Vieira, F. J., Joaquim, L. C., . . . Gustafson, P. (2011). Impact of tuberculosis exposure at home on mortality in children under 5 years of age in Guinea-Bissau. Thorax, 66(2), 163-167. doi:10.1136/thx.2010.141309 | 1 |
| Govender, T., Barnes, J. M., & Pieper, C. H. (2010). Living in low-cost housing settlements in cape town, South Africa-the epidemiological characteristics associated with increased health vulnerability. J Urban Health, 87(6), 899-911. doi:10.1007/s11524-010-9502-0 | 2 |
| Grande, K. M., Hunter, P., Biedrzycki, P. A., & Swain, G. R. (2014). Social determinants of health in public health practice: case study of rent stipends to augment tuberculosis cluster management. J Health Care Poor Underserved, 25(4), 1799-1809. doi:10.1353/hpu.2014.0166 | 1 |
| Guo Y, Berrang-Ford L, Ford J, et al. Seasonal prevalence and determinants of food insecurity in Iqaluit, Nunavut. Int J Circumpolar Health 2015; 74: 27284. | 2 |
| Haddad S, Mohindra KS, Siekmans K, Mak G, Narayana D. “Health divide” between Indigenous and non-Indigenous populations in Kerala, India: population based study. BMC Public Health 2012; 12: 390. | 1 |
| Hanekom, M., Streicher, E. M., Van de Berg, D., Cox, H., McDermid, C., Bosman, M., . . . Warren, R. M. (2013). Population Structure of Mixed Mycobacterium tuberculosis Infection Is Strain Genotype and Culture Medium Dependent. PLoS One, 8(7). doi:10.1371/journal.pone.0070178 | 4 |
| Hino, P., Takahashi, R. F., Bertolozzi, M. R., & Egry, E. Y. (2011). The health needs and vulnerabilities of tuberculosis patients according to the accessibility, attachment and adherence dimensions. Rev Esc Enferm USP, 45 Spec No 2, 1656-1660. doi:10.1590/s0080-62342011000800003 | 5 |
| Hirsch-Moverman, Y., Shrestha-Kuwahara, R., Bethel, J., Blumberg, H. M., Munguia, G., Venkatappa, T., . . . Horsburgh, C. (2011). Reasons for non-completion of latent tuberculosis infection (LTBI) treatment; a prospective cohort study in the u.s. and canada. American Journal of Respiratory and Critical Care Medicine, 183(1). Retrieved from https://www.embase.com/search/results?subaction=viewrecord&id=L70849205&from=export | 3 |
| Hoseinpoor, R., Karami, M., Mohammadi, Y., & Soltanian, A. (2017). Evaluation of Active Case Finding (ACF) of Tuberculosis in Slums Population in North of Iran. International Journal of Pediatrics-Mashhad, 5(5), 4867-4875. doi:10.22038/ijp.2017.21977.1837 | 1 |
| Ishaq, S. L., Rapp, M., Byerly, R., McClellan, L. S., O'Boyle, M. R., Nykanen, A., . . . Tavalire, H. F. (2019). Framing the discussion of microorganisms as a facet of social equity in human health. Plos Biology, 17(11). doi:10.1371/journal.pbio.3000536 | 1 |
| Jacobs, D. E. (2011). Housing-Related Health Hazards: Assessment and Remediation. In J. O. Nriagu (Ed.), Encyclopedia of Environmental Health (pp. 76-94). Burlington: Elsevier. | 9 |
| Jafta, N., Barregard, L., Jeena, P. M., & Naidoo, R. N. (2017). Indoor air quality of low and middle income urban households in Durban, South Africa. Environ Res, 156, 47-56. doi:10.1016/j.envres.2017.03.008 | 1 |
| Jafta, N., Jeena, P. M., Barregard, L., & Naidoo, R. N. (2019). Association of childhood pulmonary tuberculosis with exposure to indoor air pollution: a case control study. BMC Public Health, 19. doi:10.1186/s12889-019-6604-9 | 1 |
| Jain, R., & Rao, B. (2019). Role of laboratory services in primary health center (PHC) outpatient department performance: an Indian case study. Primary Health Care Research and Development, 20. doi:10.1017/s1463423619000537 | 2 |
| Jiamsakul, A., Lee, M. P., Nguyen, K. V., Merati, T. P., Cuong, D. D., Ditangco, R., . . . Law, M. (2018). Socio-economic status and risk of tuberculosis: a case-control study of HIV-infected patients in Asia. Int J Tuberc Lung Dis, 22(2), 179-186. doi:10.5588/ijtld.17.0348 | 2 |
| Jones, P. (2020). Belfast Corruption 1921-1968 and the Curious Case of Ann Copeland. Urbanities-Journal of Urban Ethnography, 10, 81-94. Retrieved from <Go to ISI>://WOS:000518454800006 | 1 |
| Kajal, N. C., Bhushan, B., Aggarwal, R., Gupta, S., & Duggal, S. (2015). SOCIO-DEMOGRAPHIC PROFILE AND BASAL METABOLIC INDEX CHARACTERISTICS OF PATIENTS WITH PULMONARY TUBERCULOSIS AND THEIR TREATMENT OUTCOME IN MEDICAL COLLEGE HOSPITAL, AMRITSAR, INDIA. Journal of Evolution of Medical and Dental Sciences-Jemds, 4(20), 3462-3472. doi:10.14260/jemds/2015/500 | 3 |
| Kapoor, A. K., & Singh, K. (2016). Demographic Dynamics in Tuberculosis Patients of Delhi. International Journal of Medical Research & Health Sciences, 5(4), 43-49. Retrieved from <Go to ISI>://WOS:000375993600007 | 3 |
| Kapoor, A. K., Deepani, V., Dhall, M., & Kapoor, S. (2016). Pattern of socio-economic and health aspects among TB patients and controls. Indian J Tuberc, 63(4), 230-235. doi:10.1016/j.ijtb.2016.09.011 | 3 |
| Katsuda, N., Hinohara, Y., Tomita, K., & Hamajima, N. (2011). Structure and roles of public health centers (hokenjo) in Japan. Nagoya J Med Sci, 73(1-2), 59-68. | 3 |
| Kazerooni, P. A., Nejat, M., Akbarpoor, M., Sedaghat, Z., & Fararouei, M. (2019). Underascertainment, underreporting, representativeness and timeliness of the Iranian communicable disease surveillance system for tuberculosis. Public Health, 171, 50-56. doi:10.1016/j.puhe.2019.03.008 | 1 |
| Kebede, D., Zielinski, C., Mbondji, P. E., Sanou, I., Kouvividila, W., & Lusamba-Dikassa, P. S. (2014). Expenditures on health research in sub-Saharan African countries: results of a questionnaire-based survey. Journal of the Royal Society of Medicine, 107, 77-84. doi:10.1177/0141076814530601 | 1 |
| Kendall, E. A., Theron, D., Franke, M. F., Van Helden, P., Victor, T. C., Warren, R. M., . . . Jacobson, K. R. (2013). Alcohol, social instability, and default from multidrug resistant tuberculosis treatment in rural South Africa. American Journal of Tropical Medicine and Hygiene, 89(5), 141. Retrieved from https://www.embase.com/search/results?subaction=viewrecord&id=L71312297&from=export | 1 |
| Kerkhoff, A. D., Muyoyeta, M., & Cattamanchi, A. (2020). Community-wide Screening for Tuberculosis. New England Journal of Medicine, 382(12), 1185-1186. doi:10.1056/NEJMc1916666 | 3 |
| Kerubo, G., Amukoye, E., Niemann, S., & Kariuki, S. (2016). Drug susceptibility profiles of pulmonary Mycobacterium tuberculosis isolates from patients in informal urban settlements in Nairobi, Kenya. BMC Infect Dis, 16(1), 583. doi:10.1186/s12879-016-1920-5 | 1 |
| Khan, M. K., Islam, M. N., Ferdous, J., & Alam, M. M. (2019). An Overview on Epidemiology of Tuberculosis. Mymensingh Med J, 28(1), 259-266. | 3 |
| Kilabuk, E., Momoli, F., Mallick, R., Van Dyk, D., Pease, C., Zwerling, A., . . . Alvarez, G. G. (2019). Social determinants of health among residential areas with a high tuberculosis incidence in a remote Inuit community. J Epidemiol Community Health, 73(5), 401-406. doi:10.1136/jech-2018-211261 | 1 |
| Kim, J. Y., Farmer, P., & Porter, M. E. (2013). Redefining global health-care delivery. Lancet, 382(9897), 1060-1069. doi:10.1016/s0140-6736(13)61047-8 | 2 |
| Kobbe, R., Kitz, C., Trapp, S., Pfeil, J., & Hufnagel, M. (2016). Care of child and adolescent refugees: Focus on diagnosis and prevention of infectious diseases. Notfall und Rettungsmedizin, 19(5), 346-354. doi:10.1007/s10049-016-0180-7 | 5 |
| Krishnan, A., Kumar, R., Nongkynrih, B., Misra, P., Srivastava, R., & Kapoor, S. K. (2012). Adult mortality surveillance by routine health workers using a short verbal autopsy tool in rural north India. Journal of Epidemiology and Community Health, 66(6), 501-506. doi:10.1136/jech.2010.127480 | 1 |
| Lai, K. M., Ozuah, Z. H., Williams, P., & Piccinini, L. S. (2012). Systems connectivity between social, cultural and economic factors and housing environments associated with TB transmission in slums. Journal of Environmental Planning and Management, 55(3), 355-367. doi:10.1080/09640568.2011.601190 | 3 |
| Lai, N. L. S., Kwok, K. Y., Wang, X. H., Yamashita, N., Liu, G. J., Leung, K. M. Y., . . . Lam, J. C. W. (2019). Assessment of organophosphorus flame retardants and plasticizers in aquatic environments of China (Pearl River Delta, South China Sea, Yellow River Estuary) and Japan (Tokyo Bay). Journal of Hazardous Materials, 371, 288-294. doi:10.1016/j.jhazmat.2019.03.029 | 1 |
| Larcombe L, Nickerson P, Singer M, et al. Housing conditions in 2 Canadian First Nations communities. Int J Circumpolar Health 2011; 70: 141.53. | 1 |
| Lee, J. E., Lee, B. J., Roh, E. Y., Kim, D. K., Chung, H. S., & Lee, C. H. (2011). The diagnostic accuracy of tuberculosis real-time polymerase chain reaction analysis of computed tomography-guided bronchial wash samples. Diagnostic Microbiology and Infectious Disease, 71(1), 51-56. doi:10.1016/j.diagmicrobio.2010.12.019 | 1 |
| Lessigiarska, I., Pajeva, I., Prodanova, P., Georgieva, M., & Bijev, A. (2012). Structure-Activity Relationships of Pyrrole Hydrazones as New Anti-Tuberculosis Agents. Medicinal Chemistry, 8(3), 462-473. doi:10.2174/1573406411208030462 | 1 |
| Leung, C. C., Hui, L., Lam, T. H., Yew, W. W., Law, W. S., & Tam, C. M. (2012). Tuberculosis increases the risk of lung cancer death in the elderly. American Journal of Respiratory and Critical Care Medicine, 185. Retrieved from https://www.embase.com/search/results?subaction=viewrecord&id=L71988453&from=export | 2 |
| Lim, L. K., Enarson, D. A., Reid, A. J., Satyanarayana, S., Cutter, J., Kyi Win, K. M., . . . Wang, Y. T. (2013). Notified tuberculosis among Singapore residents by ethnicity, 2002-2011. Public Health Action, 3(4), 311-316. doi:10.5588/pha.13.0055 | 3 |
| Lima, S., Rocha, J. V. M., de Araujo, K., Nunes, M. A. P., & Nunes, C. (2020). Determinants associated with areas with higher tuberculosis mortality rates: an ecological study. Tropical Medicine & International Health, 25(3), 338-345. doi:10.1111/tmi.13349 | 1 |
| Lin, K. S., Kyaw, C. S., Sone, Y. P., & Win, S. Y. (2017). Knowledge on Tuberculosis among the Members of a Rural Community in Myanmar. International Journal of Mycobacteriology, 6(3), 274-280. doi:10.4103/ijmy.ijmy_89_17 | 1 |
| Lin, Y., Li, L., Mi, F. L., Du, J., Dong, Y. Q., Li, Z. L., . . . Harries, A. D. (2012). Screening patients with Diabetes Mellitus for Tuberculosis in China. Tropical Medicine & International Health, 17(10), 1302-1308. doi:10.1111/j.1365-3156.2012.03069.x | 2 |
| Lindstrom, J., Carlsson, G. A., Wahlin, E., Tedgren, A. C., & Poludniowski, G. (2020). Experimental assessment of a phosphor model for estimating the relative extrinsic efficiency in radioluminescent detectors. Physica Medica-European Journal of Medical Physics, 76, 117-124. doi:10.1016/j.ejmp.2020.07.009 | 1 |
| Lohiniva, A. L., Mokhtar, A., Azer, A., Elmoghazy, E., Kamal, E., Benkirane, M., & Dueger, E. (2016). Qualitative interviews with non-national tuberculosis patients in Cairo, Egypt: understanding the financial and social cost of treatment adherence. Health Soc Care Community, 24(6), e164-e172. doi:10.1111/hsc.12280 | 1 |
| Lowther, S. A., Miramontes, R., Navara, B., Sabuwala, N., Brueshaber, M., Solarz, S., . . . Lynfield, R. (2011). Outbreak of Tuberculosis Among Guatemalan Immigrants in Rural Minnesota, 2008. Public Health Reports, 126(5), 726-732. doi:10.1177/003335491112600515 | 1 |
| Lucas, C. M., Davenport, E. J., Griffith, C. S., Paris, C. J., & Wong, T. (2013). Utilizing interferon gamma release assay tests to improve surveillance for latent tuberculosis infection in a population at risk. American Journal of Infection Control, 41(6), S127. doi:10.1016/j.ajic.2013.03.251 | 1 |
| Lucian Davis, J., Masae Kawamura, L., Chaisson, L. H., Grinsdale, J., Benhammou, J., Ho, C., . . . Cattamanchi, A. (2014). Impact of GeneXpert MTB/RIF on patients and tuberculosis programs in a low-burden setting: A hypothetical trial. American Journal of Respiratory and Critical Care Medicine, 189(12), 1551-1559. doi:10.1164/rccm.201311-1974OC | 1 |
| Luksamijarulkul, P., Suknongbung, S., Vatanasomboon, P., & Sujirarut, D. (2017). HEALTH STATUS, ENVIRONMENTAL LIVING CONDITIONS AND MICROBIAL INDOOR AIR QUALITY AMONG MIGRANT WORKER HOUSEHOLDS IN THAILAND. Southeast Asian Journal of Tropical Medicine and Public Health, 48(2), 396-406. Retrieved from <Go to ISI>://WOS:000400882800014 | 1 |
| Maassen, W., Wiemer, D., Frey, C., Kreuzberg, C., Tannich, E., Hinz, R., . . . Frickmann, H. (2017). Microbiological screenings for infection control in unaccompanied minor refugees: the German Armed Forces Medical Service's experience. Military Medical Research, 4. doi:10.1186/s40779-017-0123-8 | 1 |
| Mahecha, G. H., Arboleda, Y. M., Vanegas, C. V., & Montes, F. N. (2017). Factors associated with the development of tuberculosis in household contacts of patients with tuberculosis, Medellin 2015. Revista Ces Salud Publica, 8(1), 48-60. Retrieved from <Go to ISI>://WOS:000424562200005 | 1 |
| Malope, S., Nkabane-Nkholongo, E., Schumacher, R., Jack, B., Babich, L. P., Penti, B., & Shaw, K. (2015). Development of a family medicine specialty training program (FMSTP) in Lesotho. Annals of Global Health, 81(1), 39-40. Retrieved from https://www.embase.com/search/results?subaction=viewrecord&id=L72073543&from=export | 3 |
| Manalan, K., Sivakumaran, K., Nayagam, M., Villanueva, B., Cosgrove, C., & Dunleavy, A. (2018). A review of in-house Video Observed Therapy (VOT) for patients with active tuberculosis(TB), in a London teaching hospital. European Respiratory Journal, 52. doi:10.1183/13993003.congress-2018.PA528 | 1 |
| Masson, M., Bereczki, Z., Molnar, E., Donoghue, H. D., Minnikin, D. E., Lee, O. Y. C., . . . Palfi, G. (2015). 7000 year-old tuberculosis cases from Hungary - Osteological and biomolecular evidence. Tuberculosis, 95, S13-S17. doi:10.1016/j.tube.2015.02.007 | 7 |
| Mayosi, B. M., Lawn, J. E., van Niekerk, A., Bradshaw, D., Abdool Karim, S. S., & Coovadia, H. M. (2012). Health in South Africa: changes and challenges since 2009. Lancet, 380(9858), 2029-2043. doi:10.1016/s0140-6736(12)61814-5 | 1 |
| McLaren, Z. M., Schnippel, K., & Sharp, A. (2016). A Data-Driven Evaluation of the Stop TB Global Partnership Strategy of Targeting Key Populations at Greater Risk for Tuberculosis. PLoS One, 11(10), e0163083. doi:10.1371/journal.pone.0163083 | 1 |
| Mhimbira, F. A., Cuevas, L. E., Dacombe, R., Mkopi, A., & Sinclair, D. (2017). Interventions to increase tuberculosis case detection at primary healthcare or community-level services. Cochrane Database of Systematic Reviews(11). doi:10.1002/14651858.CD011432.pub2 | 1 |
| Miandad, M., Nawaz-ul-Huda, S., Burke, F., Azam, M., & Khan, I. (2016). Escalation of tuberculosis notification: an analysis of associated social factors. Bulletin of Geography-Socio-Economic Series, 33(33), 91-101. doi:10.1515/bog-2016-0027 | 3 |
| Mišković, N. (2011). Housing shortage and communal politics in European cities around 1900: the cases of Basel 1889 and Belgrade 1906. Stud Hist (Sahibabad), 26(1), 61-89. doi:10.1177/025764301002600103 | 2 |
| Mitku, A. A., Dessie, Z. G., Muluneh, E. K., & Workie, D. L. (2016). Prevalence and associated factors of TB/HIV co-infection among HIV Infected patients in Amhara region, Ethiopia. African Health Sciences, 16(2), 588-595. doi:10.4314/ahs.v16i2.29 | 2 |
| Mitruka, K., Oeltmann, J. E., Ijaz, K., & Haddad, M. B. (2011). Tuberculosis Outbreak Investigations in the United States, 2002-2008. Emerging Infectious Diseases, 17(3), 425-431. doi:10.3201/eid1703.101550 | 2 |
| Mocumbi, A. O., Stewart, S., Patel, S., & Al-Delaimy, W. K. (2019). Cardiovascular Effects of Indoor Air Pollution from Solid Fuel: Relevance to Sub-Saharan Africa. Curr Environ Health Rep, 6(3), 116-126. doi:10.1007/s40572-019-00234-8 | 2 |
| Molnarova, K., Hes, D., Mikolasova, G., Michalikova, L., Brnova, J., Kulkova, N., & Krcmery, V. (2014). Spectrum of infectious diseases in rural clinic for refugees and displaced population on Rwanda-dr Congo border: Analysis of 10,051 patients. American Journal of Tropical Medicine and Hygiene, 91(5), 462. Retrieved from https://www.embase.com/search/results?subaction=viewrecord&id=L71692740&from=export | 2 |
| Morano, J. P., Walton, M. R., Zelenev, A., Bruce, R. D., & Altice, F. L. (2013). Latent tuberculosis infection: screening and treatment in an urban setting. J Community Health, 38(5), 941-950. doi:10.1007/s10900-013-9704-y | 4 |
| Mwaanga, P., Silondwa, M., Kasali, G., & Banda, P. M. (2019). Preliminary review of mine air pollution in Zambia. Heliyon, 5(9), e02485. doi:10.1016/j.heliyon.2019.e02485 | 1 |
| Nagdev, K. J., Kashyap, R. S., Bhullar, S. S., Purohit, H. J., Taori, G. M., & Daginawala, H. F. (2015). Comparison of real-time PCR and conventional PCR assay using IS6110 region of Mycobacterium tuberculosis for efficient diagnosis of tuberculous meningitis and pulmonary tuberculosis. Indian Journal of Biotechnology, 14(1), 94-100. Retrieved from <Go to ISI>://WOS:000358463800013 | 1 |
| Nimesh, M., Joon, D., Pathak, A. K., & Saluja, D. (2013). Comparative study of diagnostic accuracy of established PCR assays and in-house developed sdaA PCR method for detection of Mycobacterium tuberculosis in symptomatic patients with pulmonary tuberculosis. Journal of Infection, 67(5), 399-407. doi:10.1016/j.jinf.2013.06.015 | 1 |
| Noahsen, P., & Larsen, H. L. (2020). [Health and healthcare in Greenland]. Ugeskr Laeger, 182(24). | 1 |
| Oberhelman, R. A., Soto-Castellares, G., Gilman, R. H., Castillo, M. E., Kolevic, L., Delpino, T., . . . Evans, C. A. (2015). A Controlled Study of Tuberculosis Diagnosis in HIV-Infected and Uninfected Children in Peru. PLoS One, 10(4). doi:10.1371/journal.pone.0120915 | 1 |
| Odone, A., Crampin, A. C., Mwinuka, V., Malema, S., Mwaungulu, J. N., Munthali, L., & Glynn, J. R. (2013). Association between socioeconomic position and tuberculosis in a large population-based study in rural Malawi. PLoS One, 8(10), e77740. doi:10.1371/journal.pone.0077740 | 1 |
| Ojo, O. (2012). Molecular tools for tuberculosis surveillance in southwest Nigeria: A capacity building and networking experience. Tropical Medicine and International Health, 17, 37. doi:10.1111/j.1365-3156.2011.02994_2.x | 1 |
| Oppong, J. R., Mayer, J., & Oren, E. (2015). The global health threat of African urban slums: the example of urban tuberculosis. African Geographical Review, 34(2), 182-195. doi:10.1080/19376812.2014.910815 | 1 |
| Otero, L., Shah, L., Verdonck, K., Battaglioli, T., Brewer, T., Gotuzzo, E., . . . Van der Stuyft, P. (2016). A prospective longitudinal study of tuberculosis among household contacts of smear-positive tuberculosis cases in Lima, Peru. BMC Infectious Diseases, 16. doi:10.1186/s12879-016-1616-x | 7 |
| Padmavathi, R. (2013). Indoor air pollution-A significant but neglected Environmental Risk of Respiratory Diseases among women in developing countries. Indian Journal of Physiology and Pharmacology, 57(5), 22-23. Retrieved from https://www.embase.com/search/results?subaction=viewrecord&id=L71857037&from=export | 3 |
| Palacio, J. D. L. (2017). Social determinants of health in patients whit tuberculosis Manizales - Colombia 2012 - 2014. Archivos De Medicina, 17(1), 38-53. Retrieved from <Go to ISI>://WOS:000406443000005 | 5 |
| Paquette, K., Cheng, M. P., Kadatz, M. J., Cook, V. J., Chen, W., & Johnston, J. C. (2014). Chest radiography for active tuberculosis case finding in the homeless: a systematic review and meta-analysis. International Journal of Tuberculosis and Lung Disease, 18(10), 1231-1236. doi:10.5588/ijtld.14.0105 | 4 |
| Patterson, M., Flinn, S., & Barker, K. (2018). Addressing tuberculosis among Inuit in Canada. Can Commun Dis Rep, 44(3-4), 82-85. doi:10.14745/ccdr.v44i34a02 | 1 |
| Pedroza, D., Luna-Herrera, J., Lopez-Garcia, S., & Sevilla-Gonzalez, M. D. L. L. (2015). Lipodystrophy in men living with coinfection of human immunodeficiency virus (HIV)-tuberculosis a case control study. American Journal of Respiratory and Critical Care Medicine, 191. Retrieved from https://www.embase.com/search/results?subaction=viewrecord&id=L72052603&from=export | 2 |
| Pereira, A. G. L., Medronho, R. D., Escosteguy, C. C., Valencia, L. I. O., & Magalhaes, M. (2015). Spatial distribution and socioeconomic context of tuberculosis in Rio de Janeiro, Brazil. Revista De Saude Publica, 49. doi:10.1590/s0034-8910.2015049005470 | 1 |
| Phetsuksiri, B., Klayut, W., Rudeeaneksin, J., Srisungngam, S., Bunchoo, S., Toonkomdang, S., . . . Suzuki, Y. (2020). The performance of an in-house loop-mediated isothermal amplification for the rapid detection of Mycobacterium tuberculosis in sputum samples in comparison with Xpert MTB/RIF, microscopy and culture. Revista do Instituto de Medicina Tropical de Sao Paulo, 62. doi:10.1590/s1678-9946202062036 | 4 |
| Phetsuksiri, B., Rudeeaneksin, J., Srisungngam, S., Bunchoo, S., Roienthong, D., Mukai, T., . . . Suzuki, Y. (2013). Applicability of In-House Loop-Mediated Isothermal Amplification for Rapid Identification of Mycobacterium tuberculosis Complex Grown on Solid Media. Japanese Journal of Infectious Diseases, 66(3), 249-251. doi:10.7883/yoken.66.249 | 4 |
| Pinhata, J. M. W., Cergole-Novella, M. C., Carmo, A. M. D., Silva, R., Ferrazoli, L., Sacchi, C. T., & de Oliveira, R. S. (2015). Rapid detection of Mycobacterium tuberculosis complex by real-time PCR in sputum samples and its use in the routine diagnosis in a reference laboratory. Journal of Medical Microbiology, 64, 1040-1045. doi:10.1099/jmm.0.000121 | 4 |
| Pittalis, S., Piselli, P., Contini, S., Gualano, G., Alma, M. G., Tadolini, M., . . . Girardi, E. (2017). Socioeconomic status and biomedical risk factors in migrants and native tuberculosis patients in Italy. PLoS One, 12(12). doi:10.1371/journal.pone.0189425 | 1 |
| Podobied, O. (2017). MEDICAL CARE OF UKRAINIAN DISPLACED PERSONS IN POSTWAR WEST GERMANY. Skhidnoievropeiskyi Istorychnyi Visnyk-East European Historical Bulletin(4), 100-106. doi:10.24919/2519-058x.4.111469 | 5 |
| Pontino, M. V., Brian, M. C., Orban, R., & Sancineto, A. E. (2013). Current epidemiological situation in Buenos Aires City. American Journal of Respiratory and Critical Care Medicine, 187. Retrieved from https://www.embase.com/search/results?subaction=viewrecord&id=L71981034&from=export | 3 |
| Pothukuchi, M., Nagaraja, S. B., Kelamane, S., Satyanarayana, S., Shashidhar, Babu, S., . . . Wares, F. (2011). Tuberculosis Contact Screening and Isoniazid Preventive Therapy in a South Indian District: Operational Issues for Programmatic Consideration. PLoS One, 6(7). doi:10.1371/journal.pone.0022500 | 1 |
| Potter, J. L., Inamdar, L., Okereke, E., Collinson, S., Dukes, R., & Mandelbaum, M. (2016). Support of vulnerable patients throughout TB treatment in the UK. J Public Health (Oxf), 38(2), 391-395. doi:10.1093/pubmed/fdv052 | 1 |
| Potter, J. L., Inamdar, L., Okereke, E., Collinson, S., Dukes, R., & Mandelbaum, M. (2016). Support of vulnerable patients throughout TB treatment in the UK. J Public Health (Oxf), 38(2), 391-395. doi:10.1093/pubmed/fdv052 | 1 |
| Prasad, B. M., Thapa, B., Chadha, S. S., Das, A., Babu, E. R., Mohanty, S., . . . Tonsing, J. (2017). Status of Tuberculosis services in Indian Prisons. Int J Infect Dis, 56, 117-121. doi:10.1016/j.ijid.2017.01.035 | 1 |
| Probandari, A., Mahendradhata, Y., Widjanarko, B., Alisjahbana, B., & Torg. (2017). Social multiplier effects: academics' and practitioners' perspective on the benefits of a tuberculosis operational research capacity-building program in Indonesia. Global Health Action, 10(1). doi:10.1080/16549716.2017.1381442 | 1 |
| Purty, A. J., Mishra, A. K., Chauhan, R. C., Prahankumar, R., Stalin, P., & Bazroy, J. (2019). Burden of Pulmonary Tuberculosis among Tribal Population: A Cross-sectional Study in Tribal Areas of Maharashtra, India. Indian Journal of Community Medicine, 44(1), 17-20. doi:10.4103/ijcm.IJCM_120_18 | 1 |
| Rachmawati, D. S., Nursalam, Wibowo, A., Budiarti, A., & Agustin, R. (2018). Family factors associated with quality of life in pulmonary tuberculosis patients in Surabaya, Indonesia. Indian Journal of Public Health Research and Development, 9(11), 1772-1776. doi:10.5958/0976-5506.2018.01701.1 | 1 |
| Raffo, L. A. (2017). Tuberculosis in Rosario. Approaches to a sociocultural history of the disease. Revista Medica De Rosario, 83(3), 128-132. Retrieved from <Go to ISI>://WOS:000423923300007 | 3 |
| Rahayu, S. R., Katsuyama, H., Demura, M., Katsuyama, M., Ota, Y., Tanii, H., . . . Saijoh, K. (2015). Factors associated with tuberculosis cases in Semarang District, Indonesia: case-control study performed in the area where case detection rate was extremely low. Environmental Health and Preventive Medicine, 20(4), 253-261. doi:10.1007/s12199-015-0443-9 | 1 |
| Rathod, S. D., Timæus, I. M., Banda, R., Thankian, K., Chilengi, R., Banda, A., . . . Chi, B. H. (2016). Premature adult mortality in urban Zambia: a repeated population-based cross-sectional study. BMJ Open, 6(3), e010801. doi:10.1136/bmjopen-2015-010801 | 2 |
| Reitmanova, S., & Gustafson, D. (2012). Rethinking immigrant tuberculosis control in Canada: from medical surveillance to tackling social determinants of health. J Immigr Minor Health, 14(1), 6-13. doi:10.1007/s10903-011-9506-1 | 1 |
| Reyes, M. D. J. B., Perez, M., Bongaerts, T., & Ortuno-Gutierrez, N. (2017). Successful integrated biomedical and social support to vulnerable people affected by Tuberculosis in Nicaragua. Tropical Medicine and International Health, 22, 218. doi:10.1111/(ISSN)1365-3156 | 3 |
| Ricks, P. M., Hershow, R. C., Rahimian, A., Huo, D., Johnson, W., Prachand, N., . . . Paul, W. (2015). A randomized trial comparing standard outcomes in two treatment models for substance users with tuberculosis. Int J Tuberc Lung Dis, 19(3), 326-332. doi:10.5588/ijtld.14.0471 | 4 |
| Riva, M., Larsen, C. V. L., & Bjerregaard, P. (2014). Household crowding and psychosocial health among Inuit in Greenland. International Journal of Public Health, 59(5), 739-748. doi:10.1007/s00038-014-0599-x | 1 |
| Romanyukha, A. A., Karkach, A. S., Borisov, S. E., Belilovskiy, E. M., & Sannikova, T. E. (2019). Identification of long-existing areas of tuberculosis infection in a metropolis using mathematical methods. Infektsionnye Bolezni, 17(2), 67-73. doi:10.20953/1729-9225-2019-2-67-73 | 5 |
| Rommel, A., & Ellert, U. (2016). Health and health-related behavior among people with migrant background in Germany. Internistische Praxis, 57(1), 121-136. Retrieved from https://www.embase.com/search/results?subaction=viewrecord&id=L614086450&from=export | 5 |
| Rommel, A., & Ellert, U. (2017). Health and health-related behavior among people with migrant background in Germany. Gynakologische Praxis, 41(3), 487-502. Retrieved from https://www.embase.com/search/results?subaction=viewrecord&id=L614691790&from=export | 5 |
| Ruiz-Castell M, Muckle G, Dewailly E, et al. Household crowding and food insecurity among Inuit families with school-aged children in the Canadian Arctic. Am J Public Health 2015; 105: e122.32. | 2 |
| Sadar, S., Darban-Sarokhalil, D., Irajian, G. R., Fooladi, A. A. I., Moradi, J., & Feizabadi, M. M. (2017). An evaluation study on phenotypical methods and real-time PCR for detection of Mycobacterium tuberculosis in sputa of two health centers in Iran. Iranian Journal of Microbiology, 9(1), 38-42. Retrieved from <Go to ISI>://WOS:000453393500006 | 4 |
| Sagoro, T. K. (2018). Characteristics of children who are in close contact with MDR TB patients in Persahabatan hospital. Pediatric Pulmonology, 53, S117-S118. doi:10.1002/ppul.24034 | 1 |
| Samal, J., & Dehury, R. K. (2017). Impact of a Structured Tuberculosis Awareness Strategy on the Knowledge and Behaviour of the Families in a Slum Area in Chhattisgarh, India. Journal of Clinical and Diagnostic Research, 11(3), LC11-LC15. doi:10.7860/jcdr/2017/24107.9489 | 2 |
| Samaria, J. K., & Chaubey, S. (2012). Nonpharmacological factors for the emergence of drug resistance in patients of pulmonary tuberculosis: An Indian overview. European Respiratory Journal, 40. Retrieved from https://www.embase.com/search/results?subaction=viewrecord&id=L71926189&from=export | 3 |
| San Pedro, A., Gibson, G., dos Santos, J. P. C., de Toledo, L. M., Sabroza, P. C., & de Oliveira, R. M. (2017). Tuberculosis as a marker of inequities in the context of socio-spatial transformation. Revista De Saude Publica, 51. doi:10.1590/s1518-8787.2017051006533 | 8 |
| Sanchez-Bustamante, L., Bailon, Z., Bongaerts, T., & Ortuno-Gutierrez, N. (2017). Biomedical and social support to vulnerable people affected by Tuberculosis in Guatemala: An effective Governmental and Non-Governmental partnership implementation. Tropical Medicine and International Health, 22, 218. doi:10.1111/(ISSN)1365-3156 | 3 |
| Saqib, S. E., Ahmad, M. M., Amezcua-Prieto, C., & Virginia, M. R. (2018). Treatment Delay among Pulmonary Tuberculosis Patients within the Pakistan National Tuberculosis Control Program. American Journal of Tropical Medicine and Hygiene, 99(1), 143-149. doi:10.4269/ajtmh.18-0001 | 1 |
| Sarivalasis, A., Bodenmann, P., Langenskiold, E., Lutchmaya-Flick, C., Daher, O., & Zellweger, J. P. (2013). High rate of completion of preventive therapy for latent tuberculosis infection among asylum seekers in a Swiss Canton. Swiss Med Wkly, 143, w13860. doi:10.4414/smw.2013.13860 | 1 |
| Sathenahalli, V. B., Minarey, N., Gornale, V., Kumar, R., Joshi, K., & Singh, H. P. (2015). ASSOCIATION OF TUBERCULOSIS WITH SEVERE ACUTE MALNUTRITION. Journal of Evolution of Medical and Dental Sciences-Jemds, 4(68), 11865-11870. doi:10.14260/jemds/2015/1710 | 1 |
| Scotto, G., Fazio, V., & Lo Muzio, L. (2017). Tuberculosis in the immigrant population in Italy: state-of-the-art review. Infez Med, 25(3), 199-209. | 2 |
| Sepulveda, T. M., de Almeida, A. K., Arce, G. M., & Pollak, T. R. Crisis over crisis: Covid-19 and two innovation proposals from Chile. Social Work Education. doi:10.1080/02615479.2020.1813702 | 1 |
| Shafee, M., Abbas, F., Ashraf, M., Mengal, M. A., Kakar, N., Ahmad, Z., & Ali, F. (2014). Hematological profile and risk factors associated with pulmonary tuberculosis patients in Quetta, Pakistan. Pakistan Journal of Medical Sciences, 30(1), 36-40. Retrieved from <Go to ISI>://WOS:000332130600009 | 1 |
| Shiferaw, D. A., Mekonnen, H. S., & Abate, A. T. (2019). House-hold contact tuberculosis screening adherence and associated factors among tuberculosis patients attending at health facilities in Gondar town, northwest, Ethiopia. BMC Infectious Diseases, 19(1). doi:10.1186/s12879-019-4695-7 | 2 |
| Silva, E. N., Pereira, A., de Araújo, W. N., & Elias, F. T. S. (2018). A systematic review of economic evaluations of interventions to tackle tuberculosis in homeless people. Rev Panam Salud Publica, 42, e40. doi:10.26633/rpsp.2018.40 | 4 |
| Silver, M. C., Neumann, P. J., Roberts, C. S., Sinha, A., Morais, E., Fang, S., & Ollendorf, D. A. (2020). PIN18 ARE WE FULLY CAPTURING THE SOCIAL IMPACT OF VACCINES? Value in Health, 23, S171. doi:10.1016/j.jval.2020.04.493 | 4 |
| Singh, S. K., Kashyap, G. C., & Puri, P. (2018). Potential effect of household environment on prevalence of tuberculosis in India: evidence from the recent round of a cross-sectional survey. Bmc Pulmonary Medicine, 18. doi:10.1186/s12890-018-0627-3 | 1 |
| Singh, S., Singh, J., Kumar, S., Gopinath, K., Balooni, V., Singh, N., & Mani, K. (2012). Poor Performance of Serological Tests in the Diagnosis of Pulmonary Tuberculosis: Evidence from a Contact Tracing Field Study. PLoS One, 7(7). doi:10.1371/journal.pone.0040213 | 4 |
| Stang, Mallongi, A., Dwinata, I., & Sumarni. (2020). Risk factor model for pulmonary tuberculosis occurrence in Makassar using spatial approach. Enfermeria Clinica, 30, 383-387. doi:10.1016/j.enfcli.2019.10.105 | 3 |
| Tabuchi, T., Takatorige, T., Hirayama, Y., Nakata, N., Harihara, S., Shimouchi, A., ... & Iso, H. (2011). Tuberculosis infection among homeless persons and caregivers in a high-tuberculosis-prevalence area in Japan: a cross-sectional study. BMC infectious diseases, 11(1), 22. | 1 |
| Tadesse, T., Demissie, M., Berhane, Y., Kebede, Y., & Abebe, M. (2013). The Clustering of Smear-Positive Tuberculosis in Dabat, Ethiopia: A Population Based Cross Sectional Study. PLoS One, 8(5). doi:10.1371/journal.pone.0065022 | 2 |
| Tadokera, R., Bekker, L. G., Kreiswirth, B. N., Mathema, B., & Middelkoop, K. (2020). TB transmission is associated with prolonged stay in a low socio-economic, high burdened TB and HIV community in Cape Town, South Africa. BMC Infectious Diseases, 20(1). doi:10.1186/s12879-020-4828-z | 7 |
| Taher-Ghasemi, Y., Nikokar, I., Yazdanbakhsh, A. R., Ebrahim-Saraie, H. S., Sadeghi, R. V., & Rajabi, A. (2016). Associations Between Socio-Environmental Determinants and the Risk of Pulmonary Tuberculosis in Guilan, Iran. Archives of Clinical Infectious Diseases, 11(1). doi:10.5812/archcid.30217 | 1 |
| Thomas, D., & Summers, R. H. (2018). Understanding patients' perceptions and experiences of directly observed therapy (DOT) for tuberculosis treatment within the United Kingdom. Thorax, 73, A221. doi:10.1136/thorax-2018-212555.378 | 3 |
| Thysen, S. M., Benn, C. S., Gomes, V. F., Rudolf, F., Wejse, C., Roth, A., . . . Fisker, A. (2020). Neonatal BCG vaccination and child survival in TB-exposed and TB-unexposed children: a prospective cohort study. BMJ Open, 10(2). doi:10.1136/bmjopen-2019-035595 | 1 |
| Toloba, Y., Soumare, D., Ouattara, K., Kanoute, T., Bore, O., Dolo, O., . . . Diallo, S. (2017). Respiratory diseases in black African carceral area. Revue Des Maladies Respiratoires, 34(7), 729-733. doi:10.1016/j.rmr.2016.10.878 | 5 |
| Toonkomdang, S., Phinyo, P., Phetsuksiri, B., Patumanond, J., Rudeeaneksin, J., Klayut, W., & Lin, B. C. (2020). Pragmatic accuracy of an in-house loop-mediated isothermal amplification (LAMP) for diagnosis of pulmonary tuberculosis in a Thai community hospital. PLoS One, 15(7). doi:10.1371/journal.pone.0236496 | 2 |
| Topp, S. M., Moonga, C. N., Mudenda, C., Luo, N., Kaingu, M., Chileshe, C., . . . Henostroza, G. (2016). Health and healthcare access among Zambia's female prisoners: a health systems analysis. International Journal for Equity in Health, 15. doi:10.1186/s12939-016-0449-y | 2 |
| uddin Siddiqui, E., Ejaz, K., Lone, S., & Raza, S. J. (2010). Investment in paediatric tuberculosis prevention in Pakistan: loss or gain? J Pak Med Assoc, 60(11), 897-901. | 1 |
| Uppada, D. R., Selvam, S., Jesuraj, N., Bennett, S., Verver, S., Grewal, H. M. S., . . . Grp, T. B. T. S. (2014). The tuberculin skin test in school going adolescents in South India: associations of socio-demographic and clinical characteristics with TST positivity and non-response. BMC Infectious Diseases, 14. doi:10.1186/s12879-014-0571-7 | 2 |
| Usami, O., Nakajima, C., Endo, S., Inomata, S., Kanamori, H., Hirakata, Y., . . . Hattori, T. (2015). A case of Manila type Mycobacterium tuberculosis infection in Japan. Clinical Case Reports, 3(7), 622-625. doi:10.1002/ccr3.277 | 1 |
| Van Wyk, S. S., Mandalakas, A. M., Enarson, D. A., Gie, R. P., Beyers, N., & Hesseling, A. C. (2012). Tuberculosis contact investigation in a high-burden setting: house or household? International Journal of Tuberculosis and Lung Disease, 16(2), 157-162. doi:10.5588/ijtld.11.0393 | 1 |
| Villa, T. C. S., Ruffino-Netto, A., Scatena, L. M., Andrade, R. L. P., Brunello, M. E. F., Nogueira, J. A., . . . Arakawa, T. (2011). Health services performance for TB treatment in Brazil: a cross-sectional study. Bmc Health Services Research, 11. doi:10.1186/1472-6963-11-241 | 1 |
| Wardani, D. W., Lazuardi, L., Mahendradhata, Y., & Kusnanto, H. (2014). Clustered tuberculosis incidence in Bandar Lampung, Indonesia. WHO South East Asia J Public Health, 3(2), 179-185. doi:10.4103/2224-3151.206734 | 1 |
| Wardani, D., & Wahono, E. P. (2018). Prediction Model of Tuberculosis Transmission Based on Its Risk Factors and Socioeconomic Position in Indonesia. Indian Journal of Community Medicine, 43(3), 204-208. doi:10.4103/ijcm.IJCM_60_18 | 8 |
| White, M. C., Nelson, R. W., Kawamura, L. M., Grinsdale, J., & Goldenson, J. (2012). Changes in characteristics of inmates with latent tuberculosis infection. Public Health, 126(9), 752-759. doi:10.1016/j.puhe.2012.04.009 | 1 |
| Wingfield, T., Boccia, D., Tovar, M., Gavino, A., Zevallos, K., Montoya, R., . . . Evans, C. A. (2014). Defining Catastrophic Costs and Comparing Their Importance for Adverse Tuberculosis Outcome with Multi-Drug Resistance: A Prospective Cohort Study, Peru. Plos Medicine, 11(7). doi:10.1371/journal.pmed.1001675 | 1 |
| Woldesemayat, E. M., Datiko, D. G., & Lindtjorn, B. (2014). Use of biomass fuel in households is not a risk factor for pulmonary tuberculosis in South Ethiopia. International Journal of Tuberculosis and Lung Disease, 18(1), 67-72. doi:10.5588/ijtld.12.0980 | 1 |
| Wolf, K., & Junker, E. (2018). Tuberculosis in Austria before, during, and after World War II. In (Vol. 43, pp. 86-93). | 1 |
| Wongkongdech, R., Srisaenpang, S., & Tungsawat, S. (2015). PULMONARY TB AMONG MYANMAR MIGRANTS IN SAMUT SAKHON PROVINCE, THAILAND: A PROBLEM OR NOT FOR THE TB CONTROL PROGRAM? Southeast Asian Journal of Tropical Medicine and Public Health, 46(2), 296-305. Retrieved from <Go to ISI>://WOS:000351554300015 | 1 |
| Workineh, M., Mathewos, B., Moges, B., Gize, A., Getie, S., Stendahl, O., . . . Abate, E. (2017). Vitamin D deficiency among newly diagnosed tuberculosis patients and their household contacts: a comparative cross-sectional study. Archives of Public Health, 75. doi:10.1186/s13690-017-0195-7 | 1 |
| Yassin, M. A., Datiko, D. G., Tulloch, O., Markos, P., Aschalew, M., Shargie, E. B., . . . Theobald, S. (2013). Innovative Community-Based Approaches Doubled Tuberculosis Case Notification and Improve Treatment Outcome in Southern Ethiopia. PLoS One, 8(5). doi:10.1371/journal.pone.0063174 | 2 |
| Zammarchi, L., Bartalesi, F., & Bartoloni, A. (2014). Tuberculosis in tropical areas and immigrants. Mediterr J Hematol Infect Dis, 6(1), e2014043. doi:10.4084/mjhid.2014.043 | 1 |
| Zuñiga, J. A. (2015). An integrated review of directly observed therapy for tuberculosis in Latin America. Hisp Health Care Int, 13(1), 46-54. doi:10.1891/1540-4153.13.1.46 | 2 |

Note: All citations were alphabetically ordered.
