# Supplementary material for: Inadequate housing and pulmonary tuberculosis: a systematic review
Source: BMC Public Health. 2022 Mar 30;22:622. doi: 10.1186/s12889-022-12879-6 (PMC8966856; doi:10.1186/s12889-022-12879-6)
Supplement: Supplementary file 4 — Additional file 4: Table S4_1. Results for risk of bias assessed using the JBI checklists. Table S4_2. Risk factors for TB other than housing. [file 12889_2022_12879_MOESM4_ESM.docx]

**Table S4_1. Results for risk of bias assessed using the JBI checklists**

|  | Q1 | Q2 | Q3 | Q4 | Q5 | Q6 | Q7 | Q8 | Q9 | Q10 | Q11 | Yes % | Risk |
| --- | --- | --- | --- | --- | --- | --- | --- | --- | --- | --- | --- | --- | --- |
| Cramm et al. (2011) [34] | O | O | X | X | O | O | O | O | - | - | - | 75.0 | Int. |
| Kerker et al. (2011) [18] | O | O | O | O | O | O | O | O | O | O | O | 100.0 | Low |
| Heo et al. (2012) [14] | O | O | O | O | O | X | O | O | - | - | - | 87.5 | Low |
| Bamrah et al. (2013) [10] | O | O | O | O | O | O | O | O | O | O | O | 100.0 | Low |
| Feske et al. (2013) [11] | O | O | O | O | O | O | O | O | O | O | - | 100.0 | Low |
| Haider et al. (2013) [24] | O | O | O | O | O | O | O | O | O | O | - | 100.0 | Low |
| Lee et al. (2013) [16] | O | O | O | O | O | O | O | O | - | - | - | 100.0 | Low |
| Ephrem et al. (2015) [33] | O | X | O | O | O | O | O | O | X | O | - | 80.0 | Low |
| Hirsch-Moverman et al. (2015) [12] | O | O | O | O | O | O | O | O | O | O | O | 100.0 | Low |
| Tesema et al. (2015) [31] | O | X | X | O | O | O | O | O | O | O | - | 80.0 | Low |
| Choi et al. (2016) [20] | O | O | O | O | O | O | O | O | O | O | O | 100.0 | Low |
| Dawson et al. (2016) [17] | O | O | O | O | O | O | O | O | - | - | - | 100.0 | Low |
| Khan et al. (2016) [29] | O | O | O | O | O | O | O | O | O | O | - | 100.0 | Low |
| Yamin et al. (2016) [9] | O | O | O | O | O | O | O | O | O | O | O | 100.0 | Low |
| Arnold et al. (2017) [13] | O | O | O | O | O | O | O | O | O | O | O | 100.0 | Low |
| Irfan et al. (2017) [27] | O | O | O | O | O | O | O | O | X | O | - | 90.0 | Low |
| Rao et al. (2018) [21] | O | O | O | O | O | O | O | O | - | - | - | 100.0 | Low |
| Kim et al. (2019) [19] | O | O | O | O | O | O | O | O | O | O | - | 100.0 | Low |
| Saqib et al. (2019) [23] | O | O | X | O | O | O | O | O | - | - | - | 87.5 | Low |
| Shimeles et al. (2019) [30] | O | O | O | O | O | O | O | O | O | O | - | 100.0 | Low |
| Wardani et al. (2019) [22] | X | X | O | O | O | X | X | O | X | O | - | 50.0 | Int. |
| Biru et al. (2020) [32] | O | O | O | O | O | O | O | O | X | O | - | 90.0 | Low |
| Kerr et al. (2020) [15] | O | O | O | O | O | O | O | O | - | - | - | 100.0 | Low |

Note: Depending on the study design, the number of questions on the JBI checklist differs; “O” and “X” refer to “Yes” and “No”, respectively. “-“ means that there are no corresponding questions. “Int.” means the intermediate level of risk.

**Table S4_2. Risk factors for TB other than housing**

|  | Socio-demographic factors | Economic factors | Incarcerated history | Smoking | Nutritional status | HIV/  AIDS | DM | Mental health | Substance use | Other chronic medical condition |  |  |
| --- | --- | --- | --- | --- | --- | --- | --- | --- | --- | --- | --- | --- |
| Cramm et al. (2011) [34] | O | O |  |  |  |  |  |  |  |  |  |  |
| Kerker et al. (2011) [18] | O |  |  |  |  | O |  |  | O | O |  |  |
| Heo et al. (2012) [14] | O |  |  | O | O |  | O |  | O | O |  |  |
| Bamrah et al. (2013) [10] | O |  | O |  |  | O |  |  | O |  |  |  |
| Feske et al. (2013) [11] | O |  | O | O |  | O |  |  | O | O |  |  |
| Haider et al. (2013) [24] | O | O | O | O |  |  |  |  |  | O |  |  |
| Lee et al. (2013) [16] | O |  |  | O | O |  | O |  |  |  |  |  |
| Ephrem et al. (2015) [33] | O | O | O | O |  | O |  |  | O | O |  |  |
| Hirsch-Moverman et al. (2015) [12] | O | O |  |  |  |  |  |  | O |  |  |  |
| Tesema et al. (2015) [31] | O | O |  | O | O |  |  |  | O |  |  |  |
| Choi et al. (2016) [20] | O | O |  | O | O |  | O |  | O |  | |  |
| Dawson et al. (2016) [17] | O | O | O |  |  | O |  | O | O |  | |  |
| Khan et al. (2016) [29] | O | O |  | O |  |  |  |  |  |  |  |  |
| Yamin et al. (2016) [9] | O | O |  | O |  | O | O | O | O |  |  |  |
| Arnold et al. (2017) [13] | O |  | O |  |  | O |  |  |  |  |  |  |
| Irfan et al. (2017) [27] | O | O |  | O |  |  | O |  |  | O |  |  |
| Pedro et al. (2017) [28] | O | O |  |  |  |  |  |  |  |  |  |  |
| Rao et al. (2018) [21] | O | O |  | O | O |  |  |  | O |  |  |  |
| Kim et al. (2019) [19] | O |  |  | O |  |  | O |  | O |  |  |  |
| Saqib et al. (2019) [23] | O | O |  | O | O |  | O |  |  | O |  |  |
| Shimeles et al. (2019) [30] | O | O |  | O |  |  |  |  |  | O |  |  |
| Wardani et al. (2019) [22] |  |  |  |  |  |  |  |  |  |  |  |  |
| Biru et al. (2020) [32] | O | O | O |  |  |  |  |  |  |  |  |  |
| Kerr et al. (2020) [15] | O |  | O |  |  | O |  | O | O | O |  |  |
| Sociodemographic factors: age, sex, race/ethnicity, education, marital status. Economic factors: employment, income. Incarcerated history: ever been in a prison, past hospitalization, military service. Nutritional status: body mass index, underweight grade. Mental health: all kind of mental disease. Substance use: drug or/and alcohol use, khat chewing. Other chronic medical condition: cancer, heart disease, cerebrovascular disease, chronic obstructive pulmonary disease, and hepatitis. DM, Diabetes Mellitus. | | | | | | | | | | | | |
